# The role of Traditional Chinese Medicine in the management of liver disease: targeting gut microbiome

**DOI:** 10.1186/s13020-026-01395-z

**Published:** 2026-04-14

**Authors:** Yifan Zhang, Hongkun Li, Fei Yu, Mengmeng Gao, Yang Wang, Siyu Xin, Jiating Yang, Zhiwei Wang, Lujie Xiang, Qingjing Ru, Na Jiang

**Affiliations:** 1 The Second School of Clinical Medicine, Zhejiang Chinese Medical University, Hangzhou, 310053 China; 2 Hangzhou Institute for Advanced Study, University of Chinese Academy of Sciences, Hangzhou, 310024 China; 3 The Second Affiliated Hospital of Zhejiang, Chinese Medical University, No. 318, Chao Wang Road, Hangzhou, 310005 Zhejiang People’s Republic of China

**Keywords:** Gut microbiome, Gut mucosal barrier, Indirect pharmacology, Intermediate substances, Liver disease, Natural products, Traditional Chinese Medicine

## Abstract

In recent years, liver disease have become a major contributor to global morbidity and mortality, with current clinical treatments often limited by late diagnosis and rapid progression. This review aims to explore the underexplored role of gut microbiota (GM) in liver disease pathogenesis, and to highlight how Traditional Chinese Medicine (TCM) offers a novel therapeutic approach through indirect modulation of the GM. While conventional pharmacological strategies focus on direct organ-targeting interventions, TCM emphasizes a holistic, multi-target approach that aligns with the complex GM-liver axis. Specifically, we examine the interactions between GM and various liver diseases (alcoholic liver disease, non-alcoholic fatty liver disease, cirrhosis, acute liver injury, autoimmune hepatitis, and hepatocellular carcinoma) and focuses on the indirect axis of "TCM-GM-liver," aiming to elucidate its scientific implications from the perspective of indirect pharmacology. Unlike current research that broadly addresses GM-derived metabolites, this review innovatively details how TCM and natural products regulate specific bacterial phyla and their representative genera to influence disease progression. In conclusion, we emphasize that GM metabolites serve as critical intermediate substances in TCM-mediated hepatoprotection. By elucidating these mechanisms, this review provides a theoretical foundation for developing microbiota-guided therapeutic strategies and advancing novel drug discovery for liver diseases.

**Graphical Abstract:**

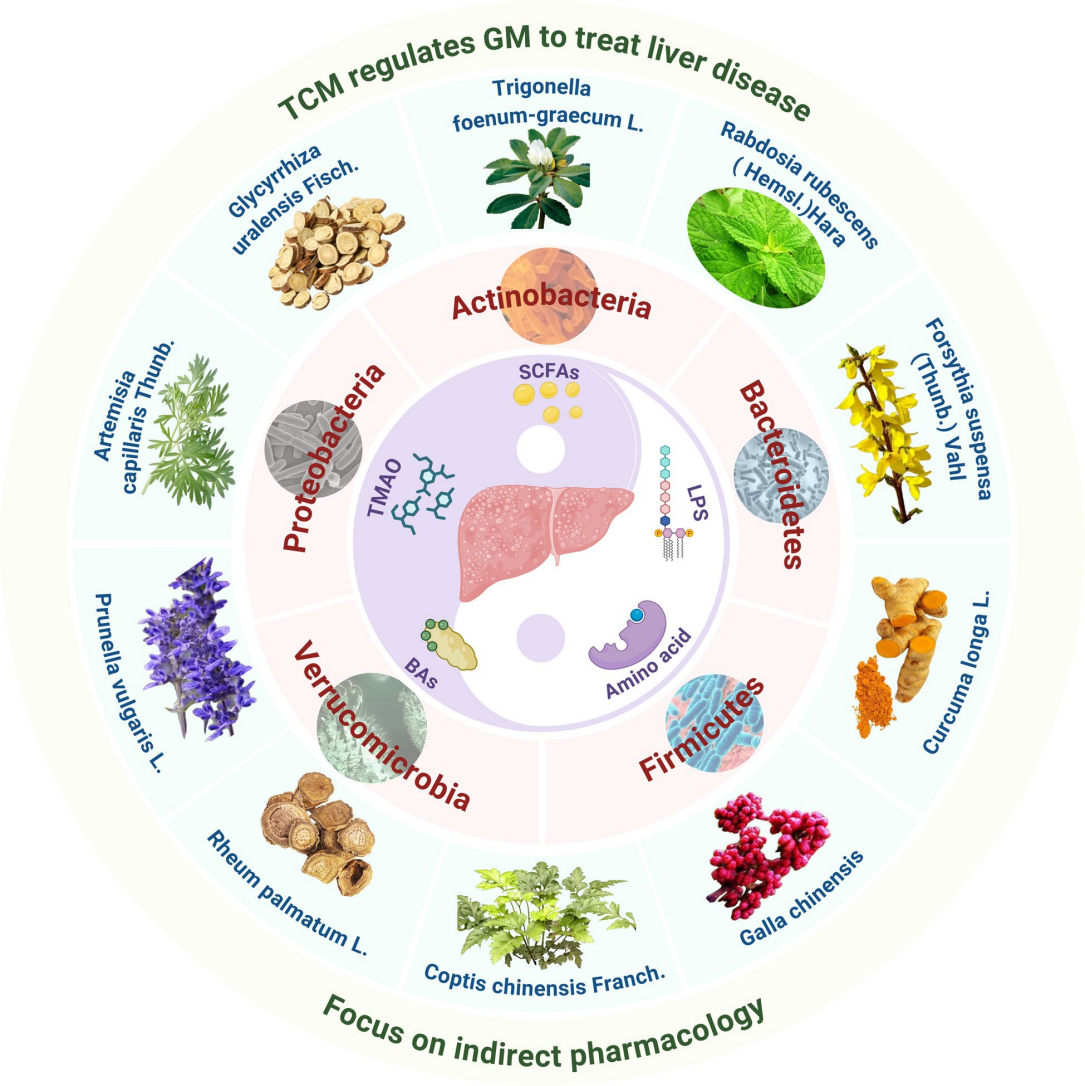

## Highlights

- The relationship between gut microbiota (GM) and clinically common liver diseases (alcoholic liver disease, non-alcoholic fatty liver disease, cirrhosis, acute liver injury, autoimmune hepatitis, and hepatocellular carcinoma) was examined.
- This article focuses on the indirect axis of "traditional Chinese medicine (TCM)-GM-liver," aiming to elucidate its scientific implications from the perspective of indirect pharmacological mechanisms.
- The mechanisms by which TCM and natural products prevent and ameliorate various liver diseases through modulation of GM, particularly the five principal phyla (Firmicutes, Bacteroidetes, Proteobacteria, Actinobacteria, and Verrucomicrobia), were summarized.

## Introduction

Epidemiological studies indicate that liver disease constitute a significant component of global morbidity and mortality, imposing a substantial economic burden and representing an urgent public health crisis [1]. Clinically common liver disorders include alcoholic liver disease(ALD), non-alcoholic fatty liver disease(NAFLD), cirrhosis, acute liver injury (ALI), autoimmune hepatitis(AIH), and hepatocellular carcinoma(HCC), among others. However, due to insufficient understanding of their pathogenesis, late-stage diagnosis, and rapid progression, clinical management strategies for liver disease still face significant challenges, directly contributing to suboptimal therapeutic outcomes [2]. Meanwhile, the theories and paradigms of traditional pharmacological research predominantly focus on the direct antagonistic effects of drugs on molecular targets in diseased organs, and continue to face challenges when addressing complex diseases such as metabolism-related disorders, autoimmune diseases, tumors, and liver diseases. Consequently, the clinical treatment and management of liver disease continue to pose considerable challenges, underscoring the pressing need for in-depth exploration of underlying mechanisms and the development of promising therapeutic agents and strategies based on such insights.

Traditional Chinese medicine (TCM), with its multi-component, multi-target, and multi-pathway pharmacological actions, emphasizes a "holistic view" and "treatment based on syndrome differentiation," aligning well with the complex mechanisms underlying liver disease. Currently, scholars have summarized and proposed that TCM pharmacology encompasses three modes of action: direct, indirect, and auxiliary [3]. This article places particular emphasis on the concept that TCM can exert clinical efficacy through indirect regulatory mechanisms. In other words, TCM acts on diseases via intermediary substances rather than through direct antagonism by its own components or their metabolites. The term "intermediary substances" here refers not to the prototype components of TCM or their metabolites, but rather to endogenous substances within the human body, such as hormones, neurotransmitters, cytokines, and exosomes, or metabolites produced by gut microbiota (GM) [4, 5]. Research has revealed that TCM exhibit cross-organ, long-range indirect regulatory effects, which can also explain numerous systemic treatment concepts in TCM, such as mutual promotion and restraint among the five zang organs, interior-exterior relationships between zang-fu organs and meridians, treating fu-organ diseases by regulating zang organs, treating child-organ diseases by addressing the mother organ, and treating upper-body disorders by managing lower-body aspects.

The human GM comprises over 100 trillion bacteria, vastly outnumbering microbial communities in any other part of the body [6]. Beyond maintaining intestinal microenvironmental homeostasis and human health, the GM plays an indispensable role in the onset and progression of various liver disease. For instance, chronic or recent excessive alcohol consumption alters intestinal permeability, increases the abundance of Gram-negative bacteria in the gut, and promotes the development and progression of ALD [7]. Gut microbiota dysbiosis, exemplified by an increased abundance of Proteobacteria and Actinobacteria coupled with a reduction in Bacteroidetes, Prevotella, and Firmicutes, accelerates the progression of NAFLD [8]. Reduction in beneficial bacteria like Lachnospiraceae and Ruminococcus, coupled with increased abundances of barrier-disrupting taxa such as Enterobacteriaceae, Staphylococcus, and Enterococcus, induces inflammatory responses and hastens the occurrence and progression of cirrhosis [9]. Recent studies have reported that elevated levels of Streptococcus, Veillonella, and Enterobacteriaceae involved in BAs metabolism are closely associated with cholestatic liver injury [10]. An elevated relative abundance of aerobic or facultative anaerobic bacteria, such as Verrucomicrobia, alongside a reduced presence of anaerobic bacteria like Synergistetes and Lentisphaerae, contributes to the worsening of AIH progression [11]. A shift in the GM, characterized by a decline in butyrate-producing genera (e.g., Ruminococcus, Oscillibacter, Faecalibacterium, and Clostridium IV) and a rise in lipopolysaccharide-producing bacteria (e.g., Klebsiella and Haemophilus), promotes HCC development [12]. Thus, targeting the GM for the treatment of various liver disease warrants further in-depth investigation.

**Fig. 1:**
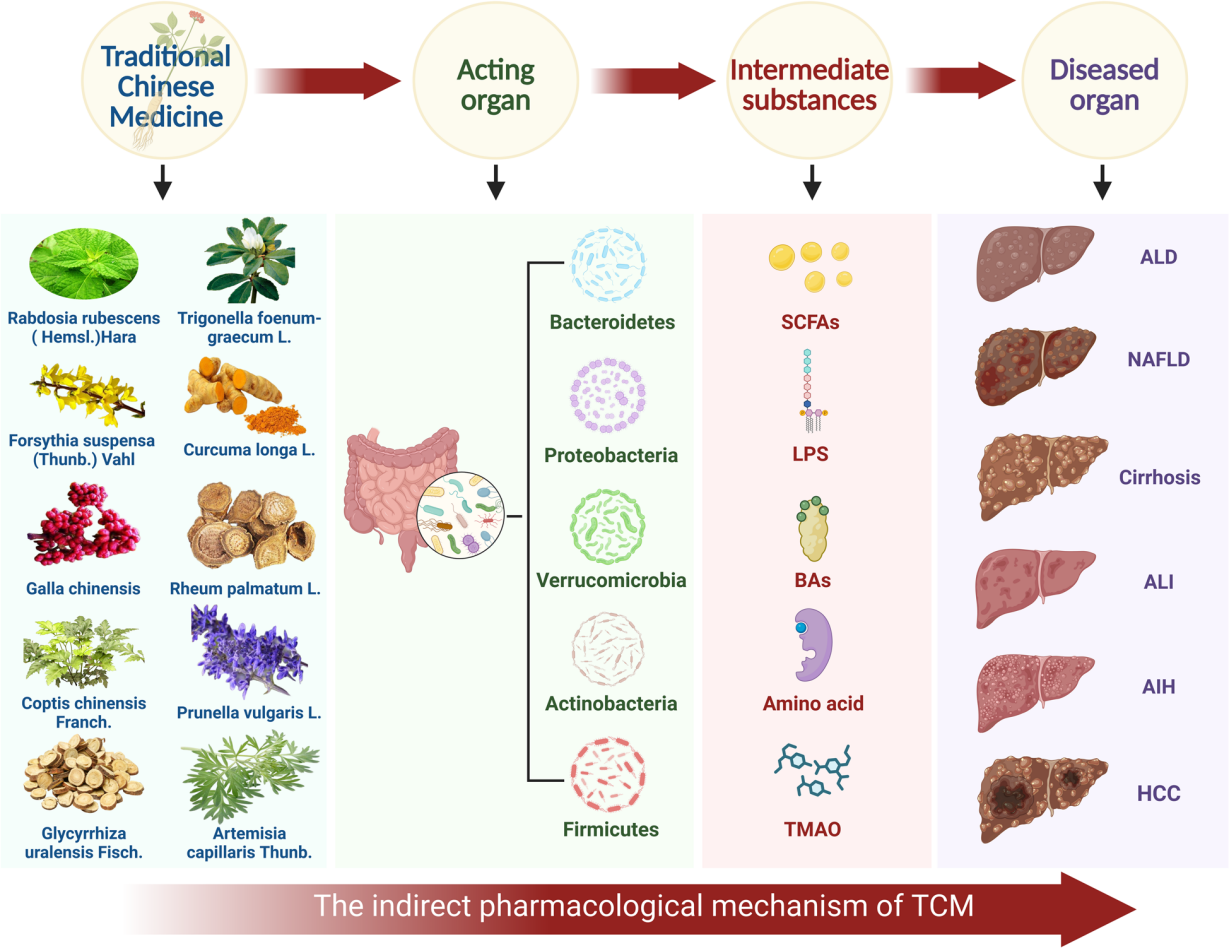
The indirect pharmacological mechanism of TCM in treating liver diseases

Currently, growing attention has been focused on the indirect axis of interaction among "TCM-GM-liver". For example, the herbal compound Bao Gan Ning Decoction (composed of Mallotus apelta (Lour.) Müll.Arg., Eupolyphagasinensis Walker, Curcuma phaeocaulis Valeton, Panax notoginseng (Burkill) F.H. Chen, Astragalus aaronii (Eig) Zohary, Salvia miltiorrhiza Bunge, and Alchornea trewioides (Benth.) Müll.Arg.) (Patent Number: ZL200610035753.1) has been shown to alleviate hepatic fibrosis by increasing the relative abundance of beneficial bacteria Lactobacillus and Bifidobacterium [13]. Active components from Dendrobium huoshanense have been demonstrated to improve lipid metabolism and intestinal barrier function by modulating GM structure, thereby mitigating liver injury [14]. A recent randomized controlled clinical trial found that Jie Du Granules (composed of Radix Actinidiae Macrospermae, Herba Salviae Chinensis, Pseudobulbus Cremastrae, and Endothelium Coreneum Gigeriae Galli) improved survival in patients with advanced HCC by reducing the abundance of Clostridium XI and Peptostreptococcaceae, which are associated with HCC incidence [15]. Additionally, research indicates that Safflower yellow, a major active constituent of Carthamus tinctorius L., can influence HCC progression by modulating the GM and enhancing hepatic immune infiltration [16]. Simultaneously, another clinical study revealed that Ling-Gui-Zhu-Gan decoction (composed of Poria cocos(Schw.)Wolf, Ramulus Cinnamomi, Rhizoma Atractylodis Macrocephalae, and Radix Glycyrrhizae) alleviates NAFLD by restructuring the impaired GM [17]. These studies highlight the substantial potential of TCM and natural products in indirectly treating liver diseases by modulating GM and its metabolites. Therefore, this article also focuses on the indirect axis of "TCM-GM-liver," aiming to elucidate its scientific implications from the perspective of indirect pharmacology (See Fig. 1).

**Fig. 2:**
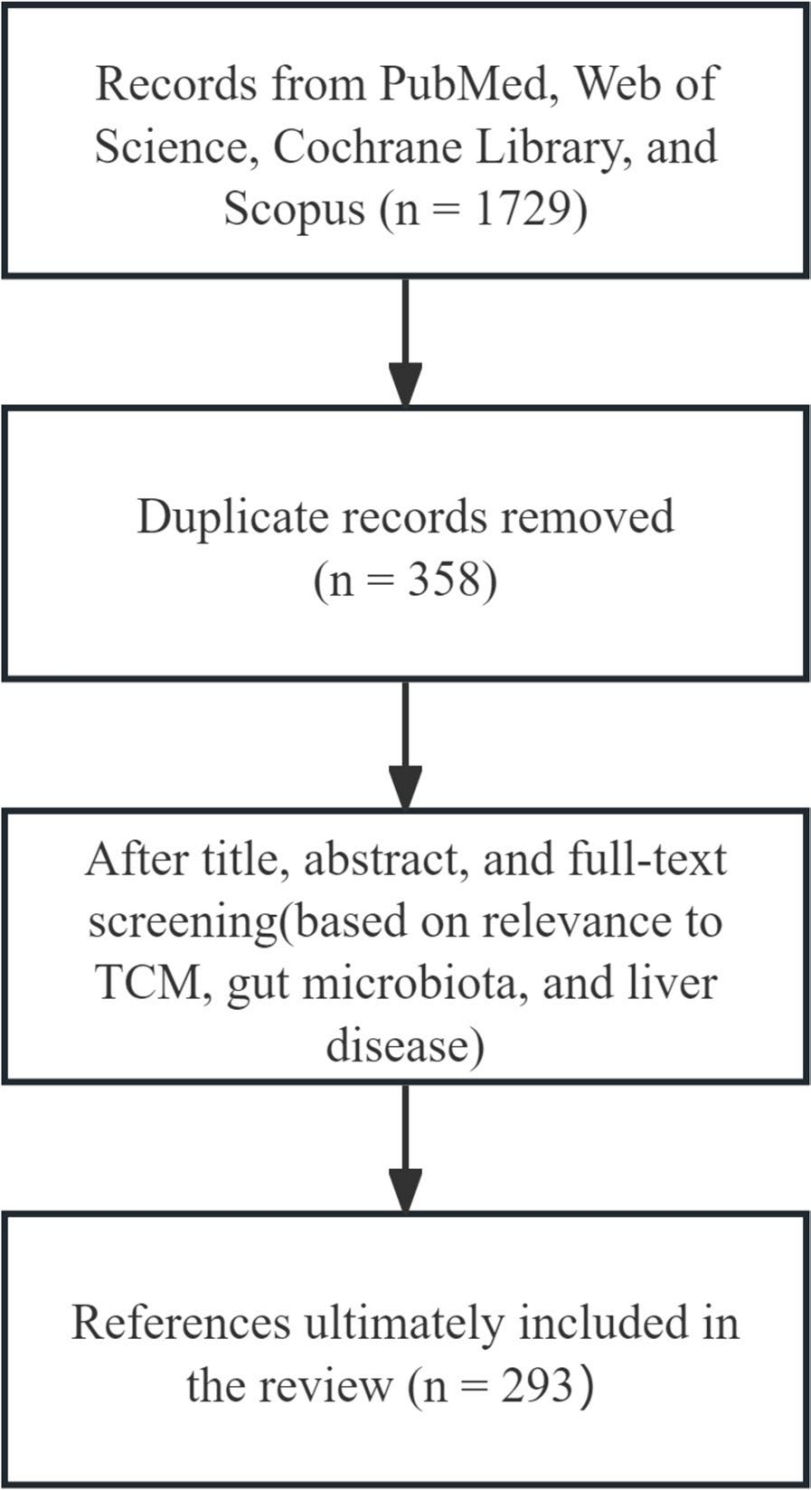
A schematic overview of the literature selection process for this review

Based on the interactions among TCM and natural products, GM, and liver disease, this review further comprehensively summarizes and explores the efficacy and mechanisms of TCM and natural products in treating liver disease by focusing on GM, particularly the five major bacterial phyla(Firmicutes, Bacteroidetes, Proteobacteria, Actinobacteria, and Verrucomicrobia) and their metabolites. In this review, we searched the PubMed, Web of Science, Cochrane Library, and Scopus databases for articles published between January 1, 2006, and February 24, 2026. The search terms included combinations of "TCM", "natural products", "GM", "indirect pharmacology", "liver disease", "ALD", "NAFLD", "cirrhosis", "ALI", "AIH" and "HCC." In addition, the reference lists of relevant articles were manually screened to identify additional studies, with priority given to high-quality original research and peer-reviewed reviews that provide mechanistic insights into the interactions among TCM, natural products, and liver pathology (See Fig. 2). This article focuses on the indirect axis of "TCM-GM-liver," aiming to elucidate its scientific implications from the perspective of indirect pharmacology: TCM and natural products modulate the structure of the GM and its metabolites, thereby indirectly influencing the progression of liver diseases, with specific emphasis on GM metabolites as key intermediary substances. It aims to provide novel perspectives and a theoretical basis for developing therapeutics and strategies against liver disease, deepen the understanding of TCM and natural products that modulate GM for liver disease treatment, and thereby facilitate their future translational research and clinical application.

## Relationship between GM and liver disease

### Alcoholic liver disease

Globally, ALD stands as a major contributor to the rising rates of preventable liver-related illness and death [18]. Harmful alcohol consumption is a risk factor for liver injury and ALD, often occurring within the context of alcohol use disorder. Ethanol-related metabolites (acetaldehyde and acetate) and oxidative stress generated during alcohol metabolism are primary contributors to ALD [19]. Currently, alcohol abstinence remains the most effective treatment for ALD. However, achieving abstinence is challenging for patients with alcohol use disorder and for those with severe ALD, disease progression may continue even after cessation of alcohol intake, with no highly effective therapeutic interventions currently available [18]. Therefore, investigating novel and effective treatment options for ALD is necessary.

**Fig. 3:**
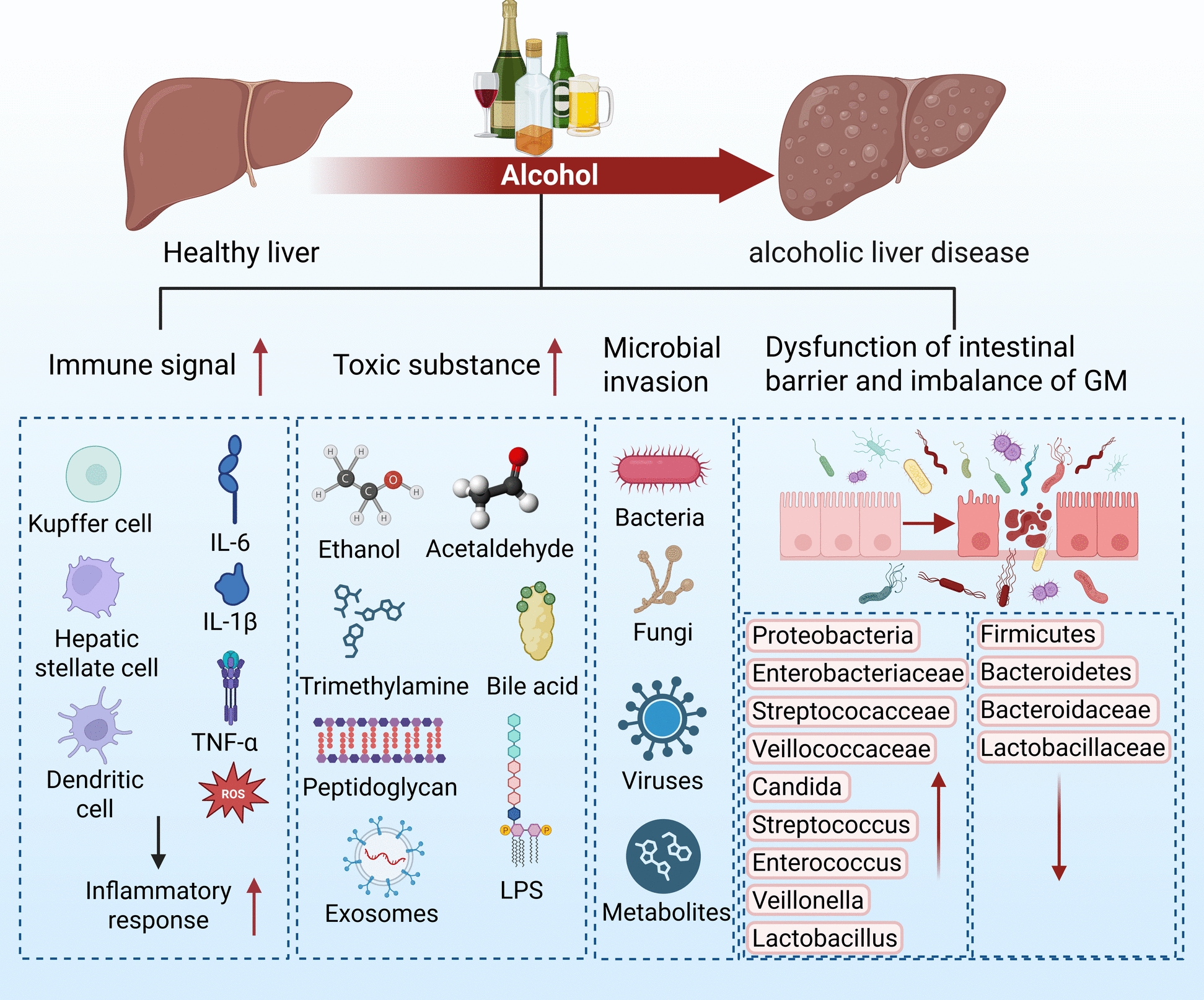
Pathophysiology of GM Dysbiosis and Microbial Characteristics in ALD. GM dysbiosis serves as the foundational element of the impaired gut-liver axis in ALD. Its salient alterations are characterized by the activation of immune signaling and toxin accumulation in the liver, concomitant with increased microbial invasion and compromised intestinal barrier integrity

Evidence implicates GM dysbiosis in driving the onset and progression of ALD (See Fig. 3) [20]. Alcohol intake initiates a cascade that begins with altering the GM. The resulting ethanol-induced increase in intestinal permeability subsequently permits bacteria and metabolites to enter circulation. This, in turn, sparks inflammation and promotes the homing of circulating immune cells to the liver, consequently affecting the functionality of hepatic immune cells[21]. Studies have found that with ALD progression, at the phylum level, the abundance of Proteobacteria increases while that of Firmicutes and Bacteroidetes decreases. At the family level, the abundance of Enterobacteriaceae, Streptococcaceae, Veillonellaceae, and Candida increases, whereas Bacteroidaceae, Lactobacillaceae, and Lachnospiraceae decrease. At the genus level, Faecalibacterium and Roseburia decrease, while Streptococcus, Enterococcus, Veillonella, and Lactobacillus increase. At the species level, the abundance of Prevotella dorei and Lactobacillus helveticus is reduced; these species can improve ALD by modulating immune cells and increasing NK cell activity [22, 23].

Multiple studies demonstrate the significant role of the GM-TLR4 axis in ALD. For instance, TLR4 deficiency in mice and intestinal sterilization using non-absorbable antibiotics (e.g.,polymyxin B、neomycin and Rifaximin [24]) both reduce hepatic steatosis, oxidative stress, and inflammation [25]. A Mendelian randomization analysis established causal links between specific GM and ALD, identifying two risk factors and one protective factor. Escherichia coli was found to exert a protective effect against ALD, whereas Roseburia hominis and bacteria from the Family Porphyromonadaceae were associated with an increased risk. Furthermore, Roseburia hominis contributes to ALD pathogenesis through its interaction with three specific inflammatory cytokines: CUB domain-containing protein 1, Cystatin D, and Monocyte chemoattractant protein-1 [26]. A randomized, double-blind, placebo-controlled study found that daily oral administration of Lactobacillus rhamnosus at a dose of 10⁹ CFU/capsule in patients with ALD significantly reduced the MELD score and the aspartate aminotransferase (AST)/alanine aminotransferase (ALT) ratio at one month, and reduced heavy drinking levels to social or abstinence levels at six months.

However, as a preliminary study that did not assess dose–response, further exploration is required [27]. In summary, under these circumstances, therapies targeting the GM show promise for the treatment of ALD.

### Non-alcoholic fatty liver disease

**Fig. 4:**
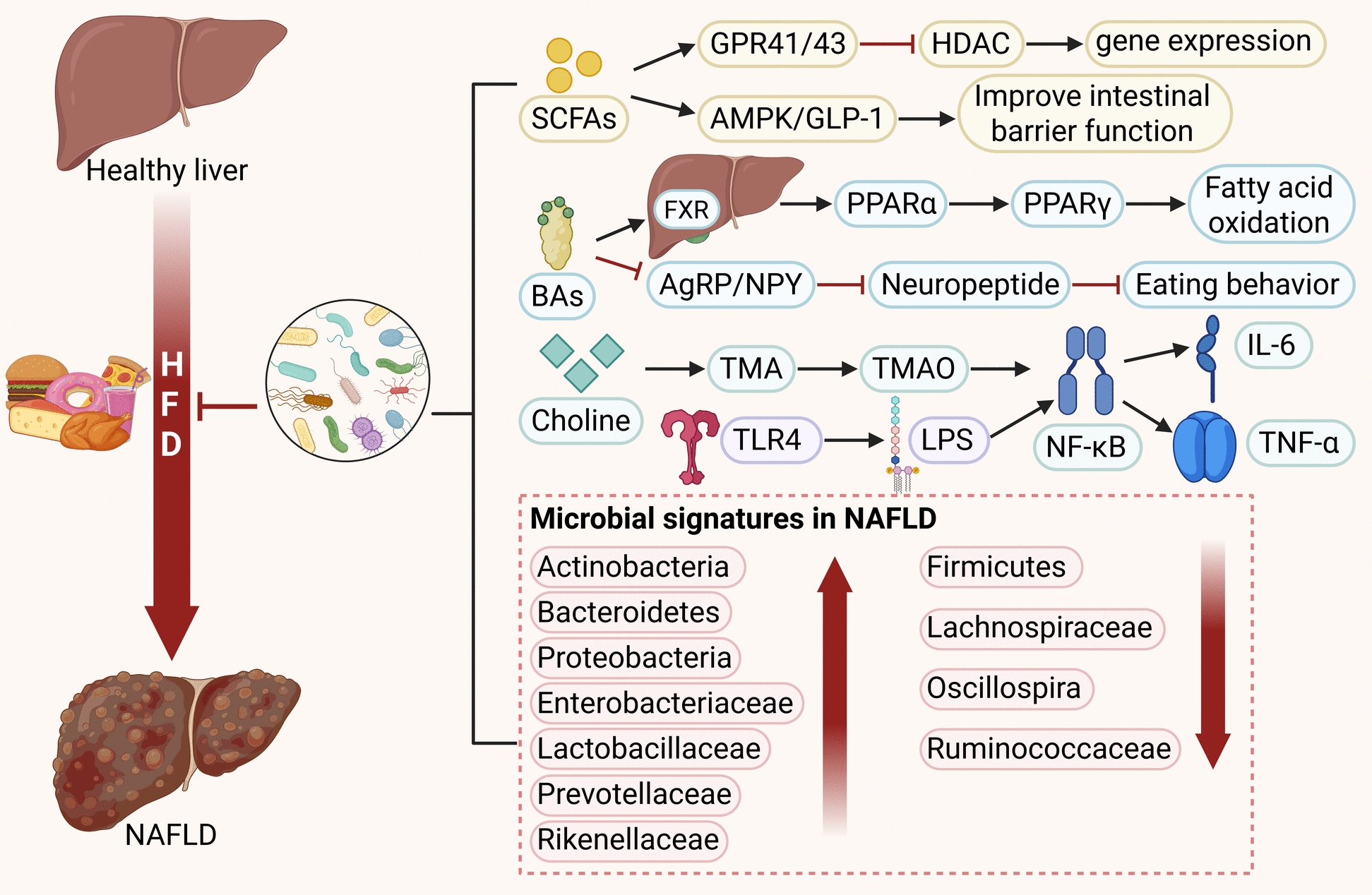
Role of GM Metabolites in the Pathogenesis of NAFLD and Gut Microbial Characteristics in NAFLD

Non-Alcoholic Fatty Liver Disease is an umbrella term encompassing a spectrum of liver conditions with varying degrees of hepatic injury and fibrosis [28]. The development NAFLD is driven by a complex interplay of factors, including dyslipidemia, impaired insulin signaling, and dysregulated adipocyte activity, along with the release of inflammatory mediators from both immune cells and adipose tissue. This pathogenic process is further influenced by oxidative and endoplasmic reticulum stress, GM dysbiosis, disruptions in genetic and epigenetic regulation, mitochondrial dysfunction, as well as various environmental and dietary determinants [8]. With the growing understanding of the GM and its regulatory mechanisms, increasing studies highlight the significant role of GM in the onset and progression of NAFLD (See Fig. 4) [29].

Alterations are observed in the GM of NAFLD patients. Specifically, at the phylum level, the abundances of Proteobacteria, Bacteroidetes, and Actinobacteria increase, while Firmicutes decreases. At the family level, the abundances of Enterobacteriaceae, Prevotellaceae, Rikenellaceae, and Lactobacillaceae increase, whereas Lachnospiraceae and Ruminococcaceae decrease [30]. Dysbiosis of the GM downregulates the expression of genes encoding tight junction proteins, which compromises intestinal barrier integrity. This impairment facilitates the translocation of pathogenic microbes and their metabolites across the intestinal epithelium. Once translocated, these components activate the immune system, inducing inflammatory responses in immune cells and thereby contributing to the progression of NAFLD [31].

Following GM dysbiosis, abnormalities in bacterial products and metabolites, such as choline, bile acids(BAs), short-chain fatty acids (SCFAs), lipopolysaccharide (LPS), and Toll-like receptor 4 (TLR4), also occur, further hastening the onset and progression of NAFLD [32]. Research indicates that in mice fed a high-fat diet, an altered gut flora metabolizes dietary choline into methylamines. This process lowers plasma phosphatidylcholine levels, which in turn compromises very low density lipoprotein secretion. The resulting reduction in hepatic lipid export contributes to the development and progression of fatty liver disease [33]. The GM also exerts direct influence on BAs metabolism. Certain bacterial species, for example, express bile salt hydrolases that transform primary BAs into secondary BAs, leading to a reduction in conjugated BAs levels. This shift has the potential to upregulate intestinal farnesoid x receptor(FXR) signaling, which may subsequently encourage the development of hepatic steatosis [34].

Disrupted production of SCFAs due to GM dysbiosis has been confirmed to be significantly associated with NAFLD progression. Metagenomic analysis reveals that decreased abundances of SCFAs-producing bacteria such as Bacteroides, Prevotella, and Roseburia correlate positively with the degree of hepatic steatosis [35]. The disruption of this microbiota-SCFAs-host axis not only affects SCFAs metabolism in the enterohepatic circulation but also exacerbates hepatic insulin resistance and lipid deposition through gut-liver axis immune crosstalk, forming a vicious cycle that drives progression from non-alcoholic steatohepatitis (NASH) to cirrhosis [36].

Numerous studies indicate that gut-derived LPS and TLR4 activation are linked to the pathogenesis of diet-induced NAFLD [37]. Clinical research further reveals a significant association between serum LPS levels, hepatic TLR4 concentration, and GM dysbiosis in NAFLD patients. Specifically, these levels positively correlate with the abundance of facultative anaerobes (e.g., Escherichia coli and Enterococcus) and negatively correlate with obligate anaerobes (including Lactobacillus, Bifidobacterium, and Bacteroides) [38]. During NAFLD progression, patients exhibit elevated serum LPS levels and increased hepatic TLR4 expression, alongside reduced GM diversity and colonization resistance. Concurrently, LPS-TLR4 signaling pathway-mediated liver injury may also contribute to NAFLD progression [39]. In summary, modulating lipid metabolism and inflammatory responses by regulating the GM represents a potential therapeutic approach for NAFLD.

### Cirrhosis

**Fig. 5:**
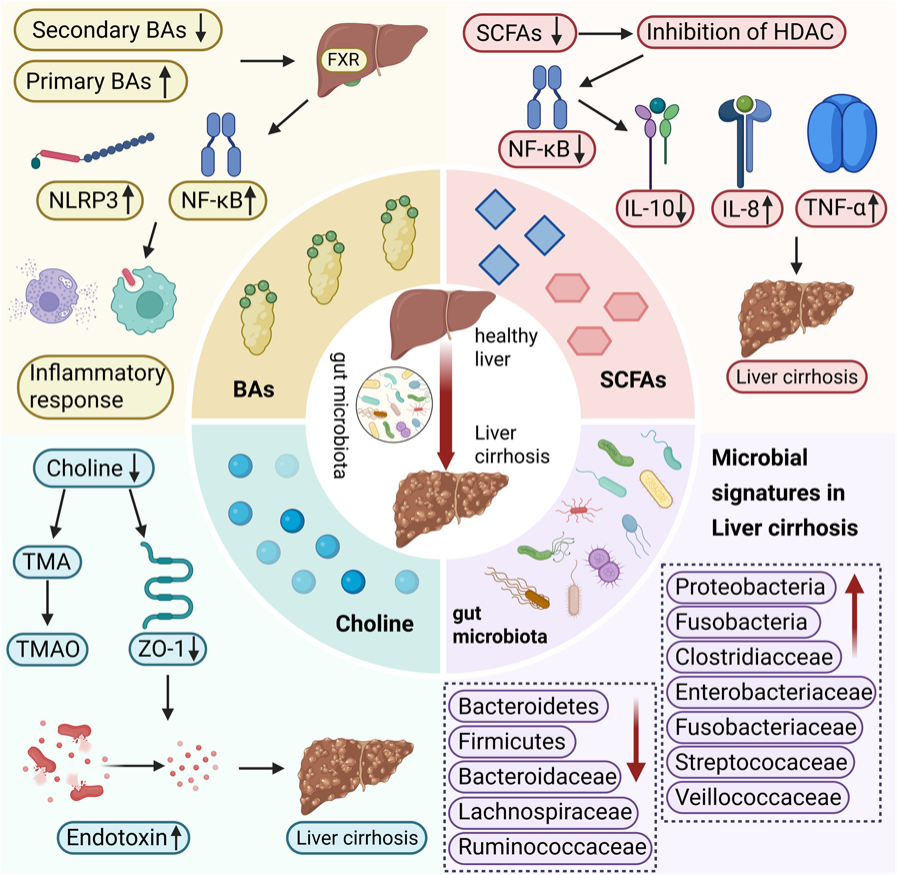
Specific Mechanisms of GM Metabolites in Cirrhosis and Gut Microbial Characteristics in Cirrhosis

Cirrhosis represents the pathological stage in which various chronic liver disease progress to feature diffuse hepatic fibrosis, pseudolobule formation, and intra- and extrahepatic vascular proliferation. It is classified into compensated and decompensated stages based on the disease's progression. Mortality in cirrhosis can reach up to 80% upon progression to the decompensated stag, as reported in a 2021 study [40, 41]. While GM dysbiosis and barrier dysfunction are integral to the pathogenesis of compensated cirrhosis, they are closely correlated with the frequency and severity of complications in decompensated cirrhosis (See Fig. 5) [22]. In decompensated patients, intestinal barrier disruption reaches an extreme, accompanied by severe microbial abnormalities and significant impairment of the intestinal physical, immune, and vascular barriers. This results in the extensive translocation of both pathogen-associated molecular patterns and viable bacteria. The consequent systemic and hepatic inflammation, along with spontaneous bacterial infections, promotes further clinical worsening and creates a self-perpetuating cycle [22, 42].

The GM alterations in cirrhosis patients often manifest as small intestinal bacterial overgrowth (SIBO). This is linked to a combination of factors such as reduced intestinal motility, lower gastric acid output, and diminished BAs secretion, all of which lead to delayed clearance of intestinal contents [43, 44]. The progression to clinical decompensation in cirrhosis is linked to SIBO. This risk is driven by microbial shifts at the phylum level, including a rise in Proteobacteria and Fusobacteria and a decline in Bacteroidetes and Firmicutes. These alterations jointly promote the buildup of metabolites and endotoxins, which in turn heighten intestinal permeability and encourage bacterial translocation [45, 46]. At the family level, abundances of Enterobacteriaceae, Streptococcaceae, Clostridiaceae, Veillonellaceae, and Fusobacteriaceae increase, while those of Bacteroidaceae, Lachnospiraceae, and Ruminococcaceae significantly decline. At the genus level, abundances of Brautia, Haemophilus, Veillonella, Dialister, and Streptococcus increase. Among these, Brautia is a major contributor to SIBO induction among all over proliferating bacteria, and its proportion serves as a key indicator reflecting cirrhosis progression [42]. Veillonella, Dialister, and Streptococcus are also identified as key components of GM dysbiosis in cirrhotic patients [9]. The activity of these bacteria, which involves producing copious neuraminidases to break down human mucin O-glycans, stimulates the release of interleukin-8 and tumor necrosis factor-alpha(TNF-α). This leads to inflammatory responses and heightened intestinal permeability, thus compromising the intestinal barrier [47, 48].

As cirrhosis advances, GM alterations drive intestinal inflammation and compromise the intestinal barrier, while also worsening hepatic inflammation; these effects collectively further inhibit hepatic BAs secretion. The subsequent reduction in intestinal FXR signaling, caused by decreased BAs, weakens the intestinal barrier by diminishing mucus thickness, lowering antimicrobial protein synthesis, and damaging the gut–vascular barrier, ultimately creating a self-reinforcing vicious cycle [49]. In summary, the GM represents a highly promising target for ameliorating cirrhosis.

### Acute liver injury

The liver performs essential physiological roles, including metabolism, synthesis, and detoxification processes [50]. It can be harmed by a range of causes, such as viral infections, adverse drug responses, heavy alcohol intake, and autoimmune disorders. Significant liver damage may result in serious complications like hepatitis, cirrhosis, and HCC. Acute liver injury is a critical disorder marked by rapid progression and elevated mortality rates [51]. In clinical practice, drug-induced liver injury (DILI) and CLI represent the most frequent forms of ALI [52].

**Fig. 6:**
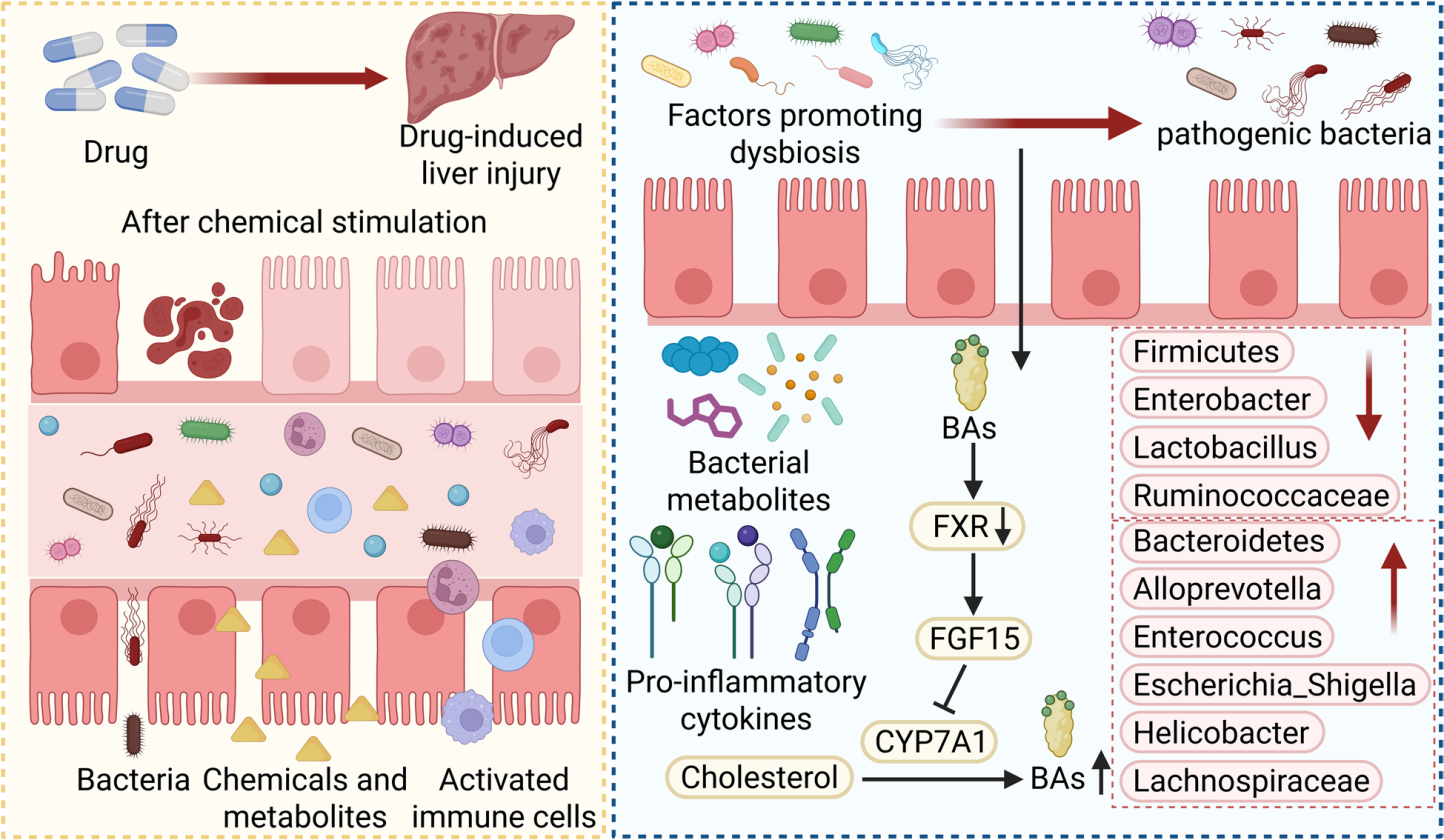
Potential Mechanisms of GM Influencing Liver Injury and Gut Microbial Characteristics in Liver Injury. Gut microbiota dysbiosis in drug-induced liver injury leads to intestinal barrier disruption, promoting the translocation of gut-derived microbes and their related metabolites, as well as the activation of immune cells. Furthermore, the GM can contribute to the development and progression of cholestatic liver injury by altering BAs metabolism and its signaling pathways

Drug-induced liver injury is the predominant form of clinical ALI in many countries, accounting for approximately 60% of cases, as reported in a 2008 study [53]. As reported in the most recent update (February 2026) of the UpToDate topic on drug-induced liver injury, this condition accounts for up to half of all acute liver failure cases in Western countries [54]. More than a thousand medications have been associated with potential hepatotoxicity. Drug-induced liver injury typically presents with a gradual and subtle onset, and its clinical features often lack specificity. The absence of established diagnostic biomarkers or an international gold standard for DILI frequently results in diagnostic delays [55–57]. Consequently, affected patients are at risk of progressing to liver failure or fatal outcomes [58]. Therefore, timely detection and treatment of DILI is particularly important. Gut microbiota dysbiosis is now widely recognized as playing a significant role in the occurrence of DILI (See Fig. 6). Among these, acetaminophen-induced liver injury has been extensively studied. Clinical data show that daily intake exceeding 4 g of acetaminophen can cause severe acute liver injury. Furthermore, the concomitant use of other drugs with acetaminophen has also been reported as a risk factor for ALI [59].

Excessive acetaminophen significantly alters the GM structure and diversity, according to 16S rRNA sequencing data. Notably, it elevates Firmicutes while reducing Bacteroidetes and Proteobacteria abundances at the phylum level. Specifically, at the genus level, a decrease is observed in both Roseburia and Lactobacillus [60]. Research has confirmed that the GM and its metabolites can mitigate acetaminophen overdose-induced DILI [61]. By increasing the abundance of the GM metabolite sedanolide, the dominant gut resident Bifidobacterium longum activates the nuclear factor erythroid 2-related factor 2 (Nrf2) pathway. This activation, in turn, confers hepatoprotection through its anti-inflammatory and antioxidant effects, inhibition of hepatocyte death, and improvement of acetaminophen-induced GM dysbiosis [60]. Concurrently, Lacticaseibacillus casei Shirota, one of the most widely used industrial lactic acid bacteria, has also been found to significantly reduce liver and ileal injury caused by acetaminophen, decrease intestinal mucosal permeability, and alleviate ALI [62]. As a common adverse effect of the first-line anti-tuberculosis drugs isoniazid and rifampicin, drug-induced liver injury can occur [63], and these agents may also induce significant and sustained dysbiosis [64]. Studies have demonstrated that administration of isoniazid and rifampicin increases the abundance of Actinobacteria at the phylum level, as well as Bacteroides and Blautia at the genus level, while decreasing the abundance of Akkermansia [65, 66]. Research has confirmed that the GM and its metabolites can alleviate drug-induced liver injury caused by isoniazid and rifampicin. For instance, Lactobacillus casei has been shown to prevent anti-tuberculosis drug-induced intestinal injury by modulating the GM and SCFAs metabolism [66]. Furthermore, a recent preclinical study revealed that the probiotic strain Bacteroides fragilis 839 ameliorates isoniazid- and rifampicin-induced liver injury by restoring GM homeostasis, enhancing intestinal barrier integrity, and attenuating inflammatory responses [67].In addition, amoxicillin-clavulanate, one of the most frequently prescribed antibiotics in Europe and the United States [68], directly disrupts the structure and diversity of the GM, leading to the depletion of beneficial bacteria and compromising hepatic protective mechanisms. This disruption is considered a major contributor to idiosyncratic drug-induced liver injury associated with oral medications [69]. A clinical study found that patients who developed idiosyncratic liver injury following amoxicillin-clavulanate administration exhibited significant enrichment of the genus Catenibacterium, along with reduced relative abundances of Barnesiella (closely associated with bile acid deconjugation metabolism) and Lachnospira (known for its anti-inflammatory potential) [70]. Supplementation with probiotics or prebiotics to eliminate harmful bacteria and restore microbial balance may represent a potential therapeutic approach for managing drug-induced liver injury [71]. These studies suggest the substantial potential of the GM as a therapeutic approach and intervention for DILI.

Cholestatic liver injury is another common form of clinical ALI, typically caused by the accumulation of BAs in the liver, and is closely linked to the GM [72]. The GM can hydrolyze the amide bond in conjugated BAs via bile salt hydrolases and generate secondary BAs through catalysis of isomerization and oxidation/reduction reactions by hydroxysteroid dehydrogenases [73]. Depletion of the GM leads to the loss of its mediated negative feedback control on BAs synthesis, reduced signaling to the FXR, elevated hepatic BAs concentrations, impaired biliary barrier function, and ultimately exacerbation of CLI [74].

16S rRNA sequencing has also shown that severe CLI significantly alters GM composition and diversity. At the phylum level, the abundance of Bacteroidetes increases while that of Firmicutes decreases. At the genus level, abundances of Alloprevotella, Enterococcus, Escherichia_Shigella, Helicobacter, and Lachnospiraceae_NK4A136_group increase, whereas those of Enterobacter, Lactobacillus, and Ruminococcaceae_UCG_014 decrease. Further research found that administration of Lactobacillus acidophilus to mice lowered total hepatic BAs, increased total fecal BAs, enriched fecal bile salt hydrolases activity to enhance BAs excretion, and thereby ameliorated CLI [75]. A randomized controlled clinical trial further corroborated this, showing that patients treated with ursodeoxycholic acid(UDCA) plus oral Lactobacillus acidophilus had significantly lower serum total BAs levels, increased fecal BAs, and lower fecal conjugated BAs content compared to those treated with UDCA alone, suggesting a beneficial effect of Lactobacillus acidophilus on liver function in cholestatic liver disease(NO: ChiCTR2200063330) [75]. Therefore, the abundance and composition of the GM can significantly alter the levels and proportions of BAs, holding promise as a novel therapeutic target for CLI.

### Autoimmune hepatitis

**Fig. 7:**
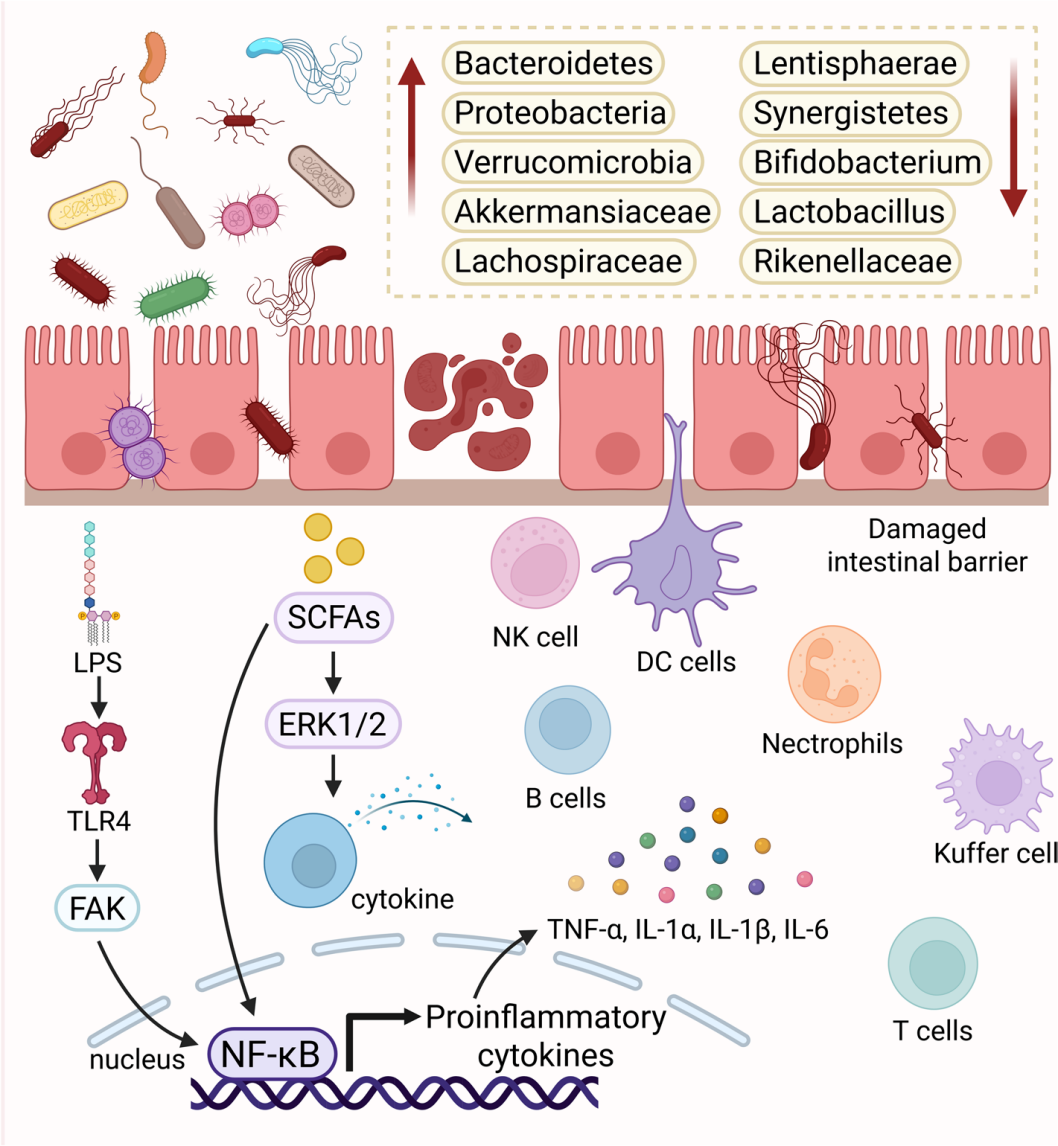
Role of GM in AIH Development and GM Characteristics in AIH. Lipopolysaccharide triggers the expression of inflammatory cytokines and amplifies immune cell responses by activating TLR4 expressed on intestinal epithelial cells, leading to the phosphorylation and activation of Focal Adhesion Kinase and consequent activation of the NF-κB signaling pathway. Meanwhile, SCFAs mediate protective immunity by activating the Extracellular Signal-Regulated Kinase 1/2 signaling pathway, thereby promoting the secretion of cytokines and chemokines

Autoimmune hepatitis is a chronic, progressive, and immune-mediated liver disease with the potential to progress to cirrhosis and liver failure [76]. To date, the pathogenesis of AIH has not been fully elucidated. Genetic susceptibility, environmental factors, and breakdown of immune tolerance are considered important contributors to its onset and progression [77]. Notably, several studies have reported that disruption of the intestinal barrier, GM dysbiosis, and dysregulation of immune homeostasis are significant factors in the development and progression of AIH (See Fig. 7) [78–80].

In a recent study, mice treated with a broad-spectrum antibiotic combination consisting of vancomycin, ampicillin, metronidazole, and neomycin, followed by transplantation with fecal microbiota from an AIH mouse model, exhibited an AIH phenotype accompanied by increased systemic autoantibodies and aggravated hepatic inflammation [79]. Compared with healthy individuals, AIH patients show significantly reduced biodiversity of the GM and an increased relative abundance of aerobic or facultative anaerobic bacteria [81, 82]. At the phylum level, the abundances of Proteobacteria, Bacteroidetes, and Verrucomicrobia are significantly increased, whereas those of Synergistetes and Lentisphaerae are markedly decreased [83]. The increase in Proteobacteria (facultative anaerobes) has been associated with inflammation, epithelial dysfunction, and disruption of host-microbiota homeostasis [78]. At the genus level, the abundances of Akkermansia and Lachnospiraceae increase, while those of Lactobacillus, Bifidobacterium, and Rikenellaceae decrease [79, 80].

Beyond the aforementioned changes in fecal microbiota, Enterococcus gallinarum is significantly enriched in the livers of AIH patients [84]. Certain specific microbial groups have also been confirmed to correlate with the severity of AIH. For instance, Veillonella exhibits the strongest association with AIH, as its abundance positively correlates with serum AST levels and the grade of liver inflammation [82]. Moreover, Roseburia, Bacteroides, Lachnospiraceae, Veillonella, and Ruminococcaceae demonstrate discriminative power between AIH patients and healthy individuals, with an average unit cost reaching 83.25% (95% CI 0.76 to 0.91) [83].

In patients with AIH, decreased GM diversity is often associated with a decline in functional genes related to SCFAs metabolism, amino acid synthesis, and vitamin metabolism. This reduction may consequently impair the host's immune regulation and metabolic processes [81]. Given that multiple factors can influence the composition and proportion of the GM, and given the discrepancies among different studies regarding the GM of AIH patients, more rigorous and long-term cohort studies are needed [85]. In summary, the GM represents a promising target for improving AIH.

### Hepatocellular carcinoma

According to recent cancer statistics, liver cancer ranks as the sixth most frequently diagnosed malignant tumor worldwide. It is also the third most common cause of death from cancer [86, 87]. Despite the established efficacy of sorafenib and regorafenib in advanced HCC, resistance frequently develops with prolonged use, adversely influencing the subsequent course of the disease [88]. Although research continues, the development of targeted therapies for HCC is still at an exploration stage and faces significant limitations in application, primarily due to the tumor's complex pathophysiology [89].

**Fig. 8:**
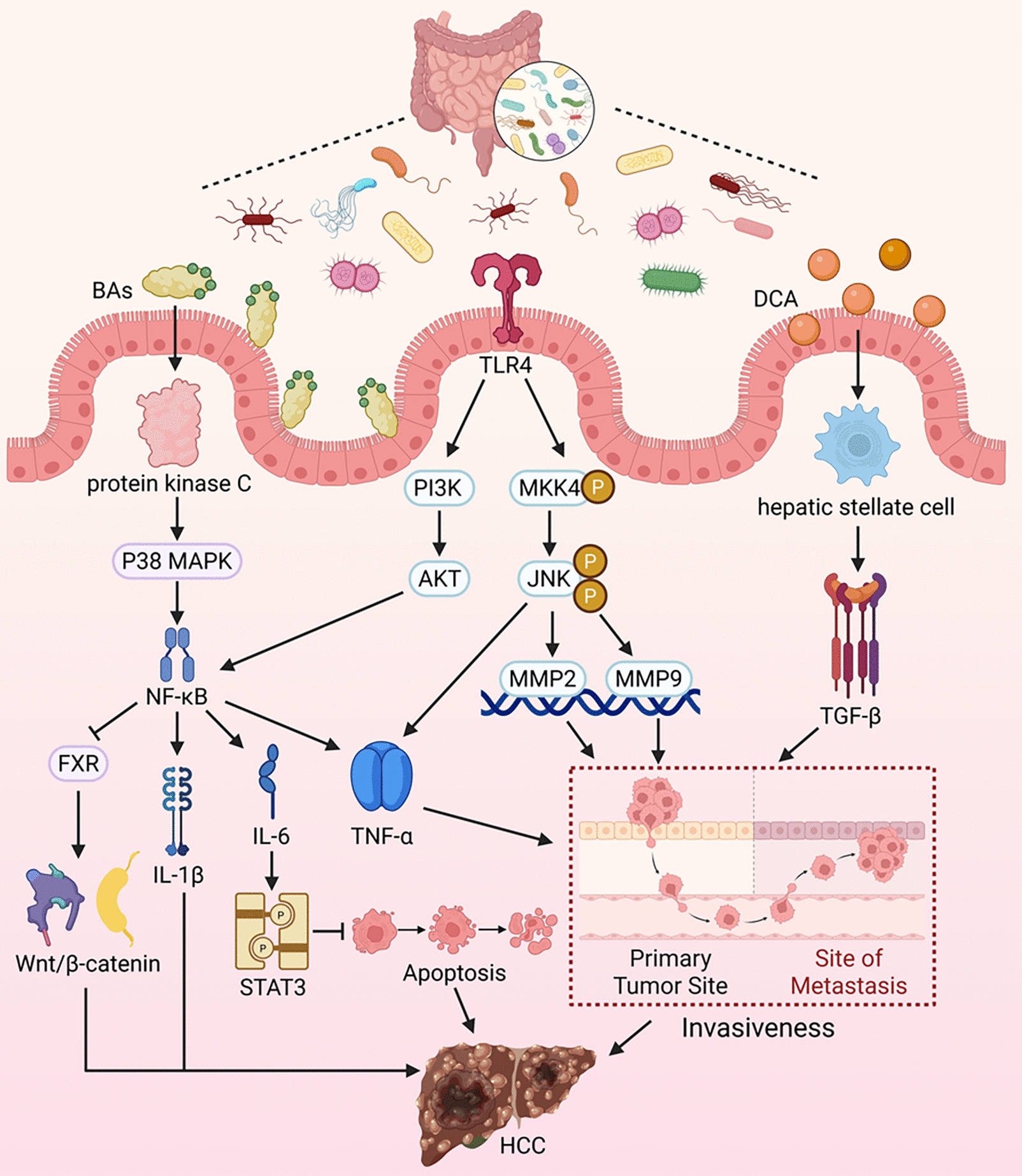
Mechanisms by Which GM Metabolites Promote HCC Development. Bile acids directly damage the plasma membrane and activate protein kinase C, which in turn activates the p38 MAPK pathway, leading to downstream activation of NF-κB. Activated NF-κB translocates to the nucleus and promotes the transcription of genes encoding Interleukin (IL)-1β, IL-6, and TNF-α. IL-6 can further influence STAT3 to inhibit apoptosis, thereby fostering HCC progression. Concurrently, NF-κB can also suppress the expression of hepatic FXR, resulting in sustained activation of the Wnt/β-catenin pathway. Toll-like receptor 4 directly activates the Mitogen-Activated Protein Kinase Kinase 4/c-Jun N-terminal Kinase signaling pathway in HCC cells, significantly enhancing the production of matrix metalloproteinase (MMP) 2, MMP9, and TNF-α, as well as increasing the invasiveness of HCC cells. Simultaneously, TLR4 can influence NF-κB by modulating the PI3K/Akt signaling pathway. Furthermore, DCA can induce the activation of quiescent hepatic stellate cells, thereby increasing the secretion of cytokines, including transforming growth factor-β (TGF-β), promoting epithelial-mesenchymal transition of cancer cells, and enhancing HCC metastasis

In recent years, studies have shown that the GM profile of HCC patients differs from that of non-HCC controls [90, 91]. For instance, at the phylum level, the abundances of Verrucomicrobia and Chytridiomycota decrease while that of Ascomycota increases. At the genus level, abundances of Bifidobacterium, Lactobacillus, Alistipes, Phascolarctobacterium, and Ruminococcus decrease, whereas those of Parabacteroides, Clostridium, Gemmiger, Klebsiella, and Haemophilus increase. These differences suggest that GM diversity may serve as a non-invasive biomarker for HCC and plays a significant role in its development (See Fig. 8) [92–94].

Furthermore, research has demonstrated that the GM and its metabolites play a crucial role in influencing various immune and metabolic events associated with HCC onset and progression [95, 96]. For example, Catenibacterium mitsuokai can disrupt the intestinal barrier, target HCC cells via the Gtr1/RagA-γ-catenin pathway, and secrete quinolinic acid to drive the PI3K/AKT pathway, thereby promoting HCC development [97]. Other studies have found that GM depletion impairs intestinal tryptophan metabolism and upregulates sterol regulatory element-binding protein 2, playing an indispensable role in promoting HCC progression [98].

Additionally, GM dysbiosis releases carcinogenic and senescence-promoting metabolites, such as deoxycholic acid (DCA), and increases hepatic exposure to LPS, which in turn promotes liver inflammation, fibrosis, proliferation, and the activation of anti-apoptotic signals, ultimately fostering HCC occurrence and development [99]. A DCA-supplemented diet increased HCC incidence in mice, whereas blocking DCA production or ameliorating intestinal bacterial dysbiosis effectively prevented HCC in obese mice [100]. In synergy with the TLR2 agonist lipoteichoic acid, DCA promotes hepatic stellate cells to adopt a senescence-associated secretory phenotype, which then suppresses anti-tumor immunity via a prostaglandin E2-dependent mechanism [101]. Given the significant contribution of the GM-liver axis to chronic liver disease progression and hepatocarcinogenesis, it represents a promising target for preventive strategies. Therefore, the prevention of HCC by targeting the GM is particularly attractive.

## Relationship between TCM and natural products and the GM

The GM is closely linked to TCM and natural products. The composition and metabolism of the GM are influenced by TCM and natural products; conversely, the GM can metabolize the medicinal components of TCM and natural products [102]. Traditional Chinese medicine and natural products exerts four primary modes of action on the GM: promotion, inhibition, eradication, and new colonization. Promotion, inhibition, and eradication directly affect the abundance of GM. Numerous studies demonstrate that TCM and natural products treatment can significantly alter the GM by promoting the growth of beneficial bacteria (e.g., those producing anti-inflammatory compounds or SCFAs) and preventing the overgrowth of harmful bacteria (e.g., pro-inflammatory and pathogenic species), thereby helping to maintain a healthy gut environment by balancing probiotic and pathogenic populations [103]. For instance, continuous intragastric administration of an aqueous extract of Ephedra sinica (165 mg/5 mL/kg) for three weeks increased the relative abundance of Blautia, Roseburia, Clostridium, and Akkermansia, subsequently influencing LPS and SCFAs levels. Simultaneously, Ephedra sinica can reduce the relative abundance of pathogenic bacteria such as Neisseria gonorrhoeae and Streptococcus mutans [104].

Additionally, the GM plays a role in modulating the absorption and metabolism of TCM and natural products, which can contribute to improved treatment outcomes and diminished adverse effects [105]. One key mechanism is the GM-mediated uptake and biotransformation of TCM constituents. For instance, dietary sources such as strawberries and raspberries contain ellagic acid, which is released in the small intestine and further metabolized by the GM before its derivatives are absorbed [106]. Moreover, following oral administration, many TCM components, especially those with low oral bioavailability like polyphenols, flavonoids, polysaccharides, and saponins, are not fully absorbed in the gastrointestinal tract[107]. Upon reaching the colon, these undigested compounds can interact with GM. Through microbial fermentation involving reactions like hydrolysis, oxidation, decarboxylation, and reduction, they are converted into smaller molecules, thereby improving oral bioavailability [108]. Consequently, the therapeutic efficacy of TCM and natural products may be enhanced via the action of the GM. Additionally, the toxicity of TCM and natural products may be reduced. For example, highly toxic diester-diterpenoid alkaloids extracted from aconite are metabolized by the GM into lipoalkaloids or lipoaconitine, which exhibit significantly lower toxicity [109]. In summary, the mechanisms by which TCM and natural products exerts its therapeutic effects are likely closely related to the GM. Meanwhile, this also demonstrates that the mechanism of action of TCM is not limited to the direct antagonistic effects on disease foci, but also encompasses cross-organ, trans-system, and remote indirect regulatory effects mediated through the modulation of intermediary substances such as GM metabolites.

## Treatment of liver disease with TCM and natural products: targeting the gut-liver axis

The human GM consists mainly of six major phyla: Firmicutes, Bacteroidetes, Proteobacteria, Actinobacteria, Verrucomicrobia, and Fusobacteria [110]. These microbial communities are essential for human health. Alterations in their composition, abundance, or diversity have been associated with the initiation and advancement of various liver disease (such as ALD [111], MAFLD [112], ALI [113], cirrhosis [114], AIH [115], and HCC [116]), ultimately impacting the host's systemic physiological balance [117]. Given that Fusobacteria constitute a minor proportion of the human GM and related research remains limited, the following section will primarily elaborate on how TCM and natural products modulate the other five major bacterial phyla and their representative genera to exert indirect therapeutic effects on liver disease by altering metabolite profiles. This analysis aims to provide a new perspective for clinically improving various hepatic disorders and to offer a theoretical basis for the development of new drugs.

### Firmicutes

The human GM is predominantly composed of two major phyla, Firmicutes and Bacteroidetes, which account for over 90% of the total community [118]. Clostridium represents a significant part of Firmicutes. Beneficial species within it, such as butyrate-producing Clostridium cluster XIVa and IV, which are also involved in bile acid metabolism, are typically reduced in abundance in ALD mice [119]. This reduction leads to impaired intestinal barrier function, allowing easier entry of bacteria and LPS from the gut into the portal vein, thereby triggering inflammation. Concurrently, disrupted BAs metabolism affecting FXR signaling promotes the onset and progression of ALD [120]. Chrysanthemum morifolium Ramat., a typical edible TCM, has been confirmed to possess anti-inflammatory, antioxidant, and hepatoprotective effects. Liu et al. reported that continuous intragastric administration of a water extract of Chrysanthemum morifolium Ramat. (2.1, 4.2, or 8.4 g/kg) for six weeks alleviated hepatic steatosis, oxidative stress, and inflammation in ALD rats via the gut–liver axis by modulating the overall structure of the GM, such as maintaining the Firmicutes/Bacteroidetes ratio and reducing the relative abundance of harmful bacteria including Clostridium and Faecalibaculum, while activating the 15d-prostaglandin J2/Peroxisome proliferator-activated receptor (PPAR)γ and PPARα signaling pathways to lower serum levels of TNF-α, IL-1β, and IL-6, thereby improving lipid metabolism [121].

Furthermore, Clostridium has been demonstrated to exert beneficial effects on intestinal barrier function by activating the aryl hydrocarbon receptor [122]. Among them, unclassified_Clostridia_UCG_014 shows a very close correlation with LPS biosynthesis substrates such as histidine tyrosine, N-palmitoyl tyrosine, and N-dihydroxy-L-valine [123]. Matrine is a natural quinazoline alkaloid used in China as a hepatoprotective agent against liver injury caused by chronic hepatitis B, with minimal side effects [124]. Additionally, Matrine has been shown to alleviate liver injury in mice exposed to carbon tetrachloride (CCl₄) and in Solt-Farber precancerous model rats [125]. Continuous administration of Matrine (100 mg/kg/d) for four weeks significantly increased the abundance of unclassified_Clostridia_UCG_014 and upregulated the expression of zonula occludens-1 (ZO-1) and occludin to enhance intestinal barrier function, thereby activating the HSP72 signaling pathway and alleviating CCl4-induced liver fibrosis via the "GM-liver-HSP72" axis [123].

Clostridium XI is also an order within Clostridium, with Clostridium difficile being its most "notorious" representative. C. difficile can produce toxins (enterotoxin A, cytotoxin B, and binary toxin) that trigger inflammatory responses in the colon [126]. C. difficile colonization also leads to a decrease in the abundance of Lachnospiraceae, Ruminococcaceae, and Bacteroides, and an increase in Enterobacteriaceae, further exacerbating GM dysbiosis and thereby influencing the occurrence and development of HCC [127]. Jiedu granule(composed of Radix Actinidiae Macrospermae, Herba Salviae Chinensis, Pseudobulbus Cremastrae, and Endothelium Coreneum Gigeriae Galli) is used clinically for preventing liver cancer, and its definite efficacy has been verified through over a decade of clinical application [128]. A recent randomized controlled clinical trial found that in patients receiving Jiedu granule treatment (twice daily for two months), the relative abundance of Clostridium XI exhibited a significant decreasing trend over time, suggesting that Jiedu granule may exert its anti-tumor effects by altering GM characteristics and effectively prolong survival in patients with advanced HCC [15].

Lactobacillus is also a member of Firmicutes, named for its ability to break down glucose and other sugars into lactic acid. Most subspecies within this genus are beneficial for gut health, aiding the body in recovering from excessive intestinal inflammation [129]. Studies have found that Lactobacillus can synergize with prednisone by enhancing its inhibitory effects on serum IL-21 levels and the proportion of Follicular helper T cells in peripheral blood mononuclear cells, showing potential in treating AIH [130]. Lactobacillus contributes to the amelioration of NAFLD through multiple mechanisms. It regulates pathways including AMP-activated protein kinase (AMPK)/Nrf2 and LPS-TLR4-NF-κB, thereby attenuating hepatic steatosis and oxidative stress. Furthermore, it influences immune function by modulating CD4^+^ T cell and Treg cell activity to suppress systemic inflammation. Through these coordinated effects on lipid metabolism and immune responses, Lactobacillus helps reduce hepatic inflammation and fat deposition, ultimately alleviating the condition [131]. Ling Nan Cai Yao Lu records that Gelsemium elegans Benth possesses hepatoprotective effects. Modern science has discovered that total alkaloids extracted from G. elegans Benth can successfully treat cirrhosis patients with ascites, with Koumine being the main alkaloid of G. elegans Benth [132]. Recent studies have found that koumine improves the composition of the GM, primarily by increasing the abundance of Lactobacillus johnsonii and Muribaculaceae, thereby upregulating heme oxygenase-1 (HO-1), B-cell lymphoma-2, and Nrf2 while downregulating Bax, NF-κB, and TLR4, and consequently mitigating Concanavalin A-induced autoimmune hepatitis via the gut-liver axis [133]. Atractylodes macrocephala extract crystallize is an active component from the dried rhizome of Atractylodes macrocephala Koidz, highly effective in regulating blood lipid levels (increasing high-density lipoprotein cholesterol (HDL-C)). Studies have found that Atractylodes macrocephala extract crystallize alleviates MAFLD via the gut-liver axis by modulating the GM, such as increasing the abundance of Lactobacillus and Muribaculaceae while reducing that of Blautia, thereby elevating HDL-C levels and decreasing LPS levels [134]. Xiayuxue decoction (XYXD), a traditional Chinese herbal formulation, is included in China's National Health Commission Guidelines for Diagnosis and Treatment of Primary Liver Cancer as a recommended therapy for this condition [135]. Recent research indicates that XYXD modulates GM by enriching Bacteroides and Lactobacillus, which stimulates bile salt hydrolase production. Concurrently, it lowers the abundance of Eubacterium, thereby inhibiting the conversion of primary to secondary BAs and elevating primary BAs levels. This shift subsequently activates hepatic natural killer T cells cells to produce interferon-γ, contributing to an immune-mediated anti-HCC effect [136]. Lactobacillus reuteri and Lactobacillus murinus both belong to the Lactobacillus genus. L. reuteri can increase the thickness of the intestinal mucus layer, promote the expression of tight junction proteins in the colonic epithelium, prevent bacterial overgrowth and invasion, thereby enhancing intestinal barrier function [137]. Lactobacillus murinus can mitigate high-fat diet-induced hyperlipidemia. This effect is associated with its ability to remodel the GM by decreasing the abundance of bacteria that produce endotoxins and degrade the mucosa, while concurrently promoting the growth of beneficial bacteria, including SCFAs producers [138]. Known for its hepatoprotective and anti-HCV properties, Corydalis saxicola Bunting is a TCM historically employed by the Zhuang ethnic group in regions such as Guangxi, China, for treating liver disorders [139]. Palmatine is the most important active ingredient in Corydalis saxicola Bunting. Recent research has found that Palmatine improves liver fibrosis by ameliorating GM dysbiosis in fibrotic rats, increasing the abundance of beneficial bacteria (such as Lactobacillus reuteri and Lactobacillus murinus) to enhance intestinal barrier function, and inhibiting hepatic inflammatory factors (LPS, TNF-α, IL-6, and IL-1β) [140].

Lachnospiraceae is also an important family within Firmicutes, considered a potentially beneficial bacterium. It can enhance the intestinal barrier by producing SCFAs (such as butyrate) and also effectively inhibit ferroptosis in hepatocytes by activating the kelch-like ECH-associated protein 1-Nrf2 antioxidant pathway, exerting hepatoprotective effects [141]. For example, studies have demonstrated that oral fecal microbiota transplantation can enrich intestinal Lachnospiraceae, alleviating acetaminophen-induced ALI [142]. Lachnospiraceae_NK4A136_group is a significant genus within Lachnospiraceae, also considered a probiotic. It can produce SCFAs and anti-inflammatory molecules with antimicrobial activity, particularly butyrate production, which helps maintain intestinal barrier integrity and suppress inflammation in mice [143, 144]. Shaoyao gancao decoction is a famous formula from "Treatise on Febrile Diseases". Research has found that Shaoyao gancao decoction can treat CCl₄-induced liver injury by increasing the abundance of SCFAs-related probiotics such as Lachnoclostridium, Lachnospiraceae_NK4A136_group, UCG-005, and Turicibacter, thereby modulating the metabolism of glutamate, citrulline, aspartate, and arginine, and improving ammonia transport and the urea cycle [145]. Schisandra chinensis (Turcz.) Baill. is a renowned TCM primarily used for liver protection. Modern research has found that Schisandrin B can alleviate glucose and lipid metabolism disorders, hepatic inflammation, and lipid peroxidation, reduce hepatic lipid deposition, and improve NAFLD progression [146]. A recent study discovered that Schisandrin B may alleviate the occurrence and development of NAFLD by modulating signaling pathways such as PPARγ, perilipin 2, phosphoenolpyruvate carboxykinase 1, ATP citrate lyase, and fatty acid desaturase 1, increasing the abundance of beneficial bacteria like Alloprevotella and Lachnospiraceae_NK4A136_group, and simultaneously increasing the expression of ZO-1 and Occludin proteins to improve intestinal barrier damage [147].

Although mechanistic studies on these drugs targeting the Firmicutes phylum for the treatment of liver diseases are advancing (See Table 1), the current breadth and depth of research remain insufficient. Existing clinical trials are characterized by small sample sizes (< 100 participants) and short durations (< 6 months). The limited sample size directly contributes to high inter-individual variability in GM composition and abundance, which constrains the generalizability of conclusions regarding the GM. Moreover, personalized treatment responses associated with baseline microbiota characteristics are rarely subjected to stratified analysis. Given the current landscape, future efforts could include large-scale, multi-center randomized controlled trials with long-term follow-up (> 12 months) and the design of personalized treatment approaches, with outcomes stratified according to baseline microbiota clusters, alongside in-depth exploration of pharmacological mechanisms.

**Table 1:** TCM and natural products improves liver disease by regulating firmicutes

Metabolite name	Source	Model	Dose	Experimental period	Results	Regulatory effects on the microbiota	Mechanism of action	References
Yin-Chen-Hao Tang	Artemisia annua L., Gardenia jasminoides J.Ellis, and Rheum Palmatum L	ALI rat	8 g/kg	10d	ALT and AST↓	Firmicutes, Gammaproteobacteria↑ Bacteroidetes, Bacteroidota↓	Regulating GM	[113]
Inulin	Cichorium intybus L	ALD mice	–	6 weeks	LPS, IL-6, IL-10, tumor necrosis factor-α and IL-17A↓	Allobaculum, Lactobacillus and Lactococcus↓ Parasutterella↑	Inhibition of LPS-TLR4 axis and regulation of GM	[148]
Curcumin	Curcuma longa L	HCC mice	700 mg/kg	2 months	Inhibiting tumor growth and weight loss	Bacteroidetes↑ Firmicutes↓	Regulating zinc homeostasis mediated by GM	[149]
Ganshuang granules	Codonopsis pilosula (Franch.) Nannf., Bupleurum chinense DC., Paeonia lactiflora Pall. Angelica sinensis (Oliv.) Diels, Poria, Aurantii Fructus, Atractylodes lancea (Thunb.) DC., Reynoutria japonica Houtt., Taraxacum mongolicum Hand.-Mazz., Prunella vulgaris L., Salvia japonica Thunb. and Peach Kernel, Turtle shell	HF mice	1.0–4.0 g/kg	5 weeks	Liver index, ALT, and aspartate transaminase↓ tight junction-associated proteins↑	Bacteroidetes↑ Firmicutes↓	Balance GM and reduce intestinal permeability	[150]
Ganoderma lucidum ethanol extracts	Ganoderma lucidum (Curtis) P. Karst	Liver fibrosis rat	120–480 mg/kg	4 weeks	AST, ALT, IL-1β, IL-6 and TNF-α↓	Ruminococcus, Lachnospiraccae↑ Corynebacteriaceae↓	Change the metabolome and GM composition, regulate the NF—κ B and TGF—β 1/Smads signaling pathways	[151]
Yinzhihuang formula	Artemisia capillaris Thunb., Gardenia jasminoides J.Ellis, Scutellaria baicalensis Georgi and Lonicera japonica Thunb	Cholestatic liver injury mice	1.35–5.4 mg/kg	14d	ALT, ALP and Liver BAs level↓ fecal BAs level↑	Firmicutes, Clostridiales, Lachnospiraceae and Bifidobacterium pseudolongum↑ Enterobacteriales, Escherichia-Shigella and Serratia↓	Restore intestinal barrier and regulate BAs excretion	[152]
Licorice	Glycyrrhiza uralensis Fisch	Liver injury rat	225–900 mg/kg	15d	ALT, AST, LDH and TBIL↓	Firmicutes, Lactobacillus↑ Proteobacteria, Escherichiam Enterococcus and Aggregatibacter↓	Regulating GM and BAs metabolism	[153]
Trigonella foenum-graecum L	Trigonella foenum-graecum L	NAFLD mice	0.26 g/kg and 0.52 g/kg	4 weeks	FBG, AUC, HbA1c, INS HOMA-IR, TC, TG, LDL-C, OxLDL, NEFA, AST, ALT, malondialdehyde(MDA), II-6, Tnf-α, II-1β and LPS↓ HOMA-ISI, GSH, Superoxide dismutase (SOD), Il-10, Claudin-1 and Occludin ↑	Faecalibaculum, Lactobacillus, Bifidobacterium and Butyricicoccus↑ unclassified_f__Lachnospiraceae↓	Improve glucose and lipid metabolism disorders, liver oxidative stress, inflammatory response, and intestinal barrier dysfunction	[154]

### Bacteroidetes

Bacteroidetes, as foundational Gram-negative anaerobic commensals in the mammalian intestinal ecosystem, play therapeutic and regulatory roles by strengthening the intestinal mucosal barrier, maintaining immunomodulatory balance, and optimizing metabolism [155]. The Gram-negative order Bacteroidales constitutes the dominant portion of the Bacteroidetes phylum and is one of the most abundant bacterial families in the mammalian GM, including Muribaculaceae, Alistipes, Bacteroidaceae, and Prevotellaceae [155]. Muribaculaceae is a dominant genus within Bacteroidetes and exhibits a syntrophic relationship with probiotics such as Bifidobacterium and Lactobacillus [156]. In recent years, Muribaculaceae has garnered significant attention for its beneficial effects on maintaining host health. For instance, it can produce SCFAs and modulate intestinal barrier function and immune responses, making it a highly promising "next-generation probiotic" [157, 158]. Studies have found a strong association between Muribaculaceae and liver disease such as cirrhosis, liver injury, and NAFLD [159].

Glaucocalyxin A is extracted from the aerial parts of the traditional Chinese herb Rabdosia japonica (Burm.f.) Hara var. glaucocalyx (Maxim.) Hara (Lamiaceae) and has been shown to possess anticoagulant, antibacterial, anti-inflammatory, antioxidant, and antitumor properties [160]. Research has found that Glaucocalyxin A can significantly improve liver fibrosis in HF mice by increasing the abundance of Muribaculaceae and Bacteroidaceae, reducing levels of AST, ALT, total bile acid (TBA), and inflammatory markers (IL-1β, TNF-α), ameliorating hepatic pathological damage and collagen deposition, and lowering apoptosis levels [161]. Furthermore, the abundance of Muribaculaceae positively correlates with hepatic ALT and AST levels and negatively correlates with hepatic SOD levels [162]. Glycyrrhiza uralensis Fisch. (GRR) is a commonly used TCM with multiple effects, including clearing heat, detoxification, alleviating pain, and harmonizing other herbs [163]. Modern pharmacological studies indicate that GRR exhibits various pharmacological activities, with hepatoprotective effects being particularly notable. GRR is widely used in TCM for treating liver disease and drug-induced liver injury[164]. Studies have demonstrated that GRR alleviates DBL-induced liver injury by modulating FXR/Nrf2–BA-related proteins and increasing the relative abundance of Muribaculaceae, thereby reducing hepatic BA accumulation and mitigating liver inflammation and oxidative stress via the gut-liver axis [165]. Cichorium glandulosum Boiss. & Huet. (CG) is a genuine medicinal material from Xinjiang. The root of CG is widely used for treating liver injury due to its hepatoprotective effects [166, 167]. Lupeol is a key triterpenoid among the active components of CG, possessing anti-inflammatory and anti-malignant tumor properties and capable of modulating the immune microenvironment [168, 169]. Research has found that lupeol alleviates MASLD by ameliorating GM, primarily through increasing the abundance of beneficial bacteria such as Muribaculaceae, Alistipes, and Akkermansia muciniphila, thereby lowering serum TC and TG levels, accelerating BA excretion, reducing BA uptake, improving intestinal inflammation, and restoring the intestinal barrier [170].

Alistipes, belonging to the family Rikenellaceae, is a genus potentially protective against certain diseases, such as liver fibrosis, liver injury, AIH, and malignant tumor progression [162, 171]. Recent research indicated that Alistipes has a protective effect; its decreased abundance was associated with an increased recurrence rate of hepatic encephalopathy when comparing the fecal microbiomes of decompensated cirrhosis patients with those with acute hepatic encephalopathy [172]. Research has found that a reduction in Alistipes is associated with the progression of cirrhosis to decompensation. This is because Alistipes can be considered a potential producer of SCFAs, which can mitigate liver injury by reducing the accumulation of macrophages and pro-inflammatory effects in the liver [173]. Their reduction contributes to such fibrotic diseases due to decreased anti-inflammatory cytokines and the inability to suppress Th17 cells. Curcuma is a TCM with effects including promoting qi and blood circulation, detoxifying, reducing swelling, and alleviating pain. It has been used for years to treat chronic liver disease. Modern pharmacology indicates its efficacy includes antitumor activity, enzyme-lowering activity, and anti-fibrotic activity [174]. Recent studies have demonstrated that Curcuma elevates the levels of Bacteroidota and Alistipes and engages in nine metabolic pathways. This activity suppresses the TLR4/NF-κB signaling pathway, leading to decreased production of downstream inflammatory factors and contributing to the amelioration of liver fibrosis [175]. Astragali Radix is a widely used tonic herb in TCM [176]. Previous studies in mice have shown that Astragaloside IV, polysaccharide extracts, and Astragalus injections can alleviate cisplatin-induced acute kidney injury [177, 178]. Recent research found that low-dose Astragali Radix intake can significantly increase the relative abundance of Alistipes, thereby increasing SCFAs and inhibiting inflammatory effects to improve intestinal barrier disruption, consequently ameliorating liver injury [162]. Alistipes also possesses the ability to modulate mucosal Treg cell production [171]. Herpetospermum caudigerum Wall. (HC) is a traditional Tibetan medicine that has been used for thousands of years in Tibet, China, to treat biliary diseases, liver disease, and indigestion [179]. To date, different extracts of HC have been shown to possess hepatoprotective activity [180]. Research found that pretreatment with the ethyl acetate extract from HC for 9 days modulates the GM (particularly upregulating Alistipes and regulating Trp metabolism), increases tryptophan and its metabolites in the kynurenine pathway, activates the indoleamine 2,3-dioxygenase/kynurenine pathway in the livers of Con A-induced AIH mice, thereby maintaining Treg-Th17 cell balance and ultimately improving AIH [181]. Sini Powder (SNP) is a famous classical TCM formula, showing significant therapeutic effects in treating stress-related diseases, particularly depression. Besides its antidepressant effects, SNP also exhibits protective roles in tumor progression, immune disorders, and GM dysbiosis [159, 182]. Recent studies have revealed that SNP treatment suppresses tumor progression in CRS-exposed tumor-bearing mice by increasing the abundance of Alistipes and Prevotella while reducing that of Lachnospira, thereby down-regulating the expression of the stress-related protein ADRB2 and the pro-inflammatory cytokines IL-1b and VEGF [159].

Prevotellaceae is a very important group of bacteria in the human and animal gut, oral cavity, and respiratory tract. Furthermore, Prevotellaceae is closely related to liver health; changes in its quantity and composition in the gut are intimately linked to the occurrence and development of various liver disease (MASLD, liver injury, ALD, HCC, etc.) [183]. Prevotellaceae holds a dual identity: on one hand, many members within the family (e.g., Prevotella copri) are often overrepresented in various liver disease, playing a "bad role" by disrupting the intestinal barrier, producing harmful metabolites, and exacerbating the condition [184]. On the other hand, there may also be certain "good members" within the family (e.g., specific strains of Prevotella bivia) that could potentially be developed into therapeutic next-generation probiotics in the future [185]. Research has found an increase in Prevotella copri and its metabolite 5-aminovaleric acid in the GM of MASLD mice promotes hepatic lipid deposition, while a decrease in Prevotella bivia abundance is observed; Prevotella bivia has shown potential in animal experiments to improve fatty liver and suppress inflammation [186]. Berberine is an isoquinoline alkaloid extracted from herbs such as Coptis chinensis Franch. and Berberis species, used clinically in China for treating bacterial diarrhea [187]. Research found that Tunicamycin administration increased the Prevotellaceae to Erysipelotrichaceae ratio in the GM of liver-injured mice, a trend reversed by Berberine treatment. Furthermore, berberine can improve metabolic disorders in the liver caused by liver injury by alleviating endoplasmic reticulum stress in hepatocytes and modulating the GM in mice [183].

Additionally, Prevotellaceae is one of the important sources of SCFAs, and a deficiency in SCFAs is positively correlated with the occurrence and development of ALD. Radix Puerariae Lobatae, the dried root of Pueraria lobata (Willd.) Ohwi from the family Fabaceae, is commonly used as a medicine or functional food in China. Studies have shown that Pueraria polysaccharides have various effects, such as anti-diabetic, antioxidant, and immunomodulatory properties [188]. Recent research found that Pueraria extract treatment increases the levels of SCFAs-producing bacteria (e.g., Bacteroides, Ruminococcus, Prevotella) in the mouse gut, thereby increasing SCFAs production, reducing liver inflammation and intestinal barrier damage, and improving ALD [189]. Studies report that Prevotellaceae is associated with microvascular invasion in oral cancer [190] and liver cancer [191], and its abundance gradually increases with the progression of HCC [192]. Xierezhuyubuxu Decoction (XRZYBXD) is derived from the modified formulation of Dahuang Zhechong Pill. Research has reported that XRZYBXD exhibits favorable efficacy in treating HCC. Specifically, it promotes HCC cell pyroptosis by ameliorating GM, primarily through increasing the beneficial bacterium Akkermansia muciniphila and reducing harmful bacteria such as Prevotellaceae and Porphyromonadaceae, thereby elevating metabolites like indole and ursolic acid while decreasing metabolites such as betaine and DL-carnitine [193].

An increasing number of TCMs and natural products have been found to alleviate various liver diseases in vitro and in vivo (See Table 2**)**, which is closely linked to their modulatory effects on the GM. However, research on translational and clinical applications remains severely insufficient. Most studies rely on 16S rRNA sequencing analysis, an approach that offers only limited analytical value for mechanistic investigations. Therefore, further development of methodologies integrated with human disease models and in vivo germ-free animal model systems—such as metabolomics and metatranscriptomic analysis—is needed to assess microbial functions and their effects on host cells, thereby enabling in-depth exploration of the underlying mechanisms.

**Table 2:** TCM and natural products improves liver disease by regulating bacteroidetes

Metabolite name	Source	Model	Dose	Experimental period	Results	Regulatory effects on the microbiota	Mechanism of action	References
Celastrol	Tripterygium wilfordii Hook. f	HCC rat	0.5 mg/kg	10 weeks	ALT, AST and ki67, mTOR phosphorylation, FXR, RXRα↓ GCDCA, UDCA, TUDCA and GUDCA ↑	Bacteroidetes, Bacteroides Fragilis ↓	Regulating GM and hepatic BAs metabolism, inducing G0/G1 phase arrest of the cell cycle, and inhibiting the progression of HCC	[194]
Pholiota adiposa	Pholiota adiposa (Batsch) P. Kumm	HCC mice	100–300 mg/kg	14d	IL-2, IFN-γ and TNF-α↑ VEGF, AST and BUN↓	Bacteroidetes, Firmicutes, Lactobacillus, Bacteroidales_S24 − 7_group_norank, Bacteroidales, Alloprevotella and Alistipes↑ Proteobacteria, Lachnospiraceae_NK4A136_group, Prevotellaceae_UCG − 001, Helicobacter and Prevotellaceae_UCG − 003↓	Regulating GM	[195]
Quercetin	Allium sativum L	HCC mice	100 mg/kg/d	28d	IL-6, IL-12a, IL-1β, TNF-α, IL-4, GM-CSF, G-CSF and TLR4↓ IL-10 and IFN-γ↑	Bacteroidota, Muribaculaceae↓ Firmicutes, Actinobacteria, Verrucomicrobiota, Dubosiella and Akkermansia↑	Regulating GM and macrophage immunity	[196]
Taohong Siwu Decoction	Prunus persica (L.) Batsch,Carthamus tinctorius L., Rehmannia glutinosa Libosch., Angelica sinensis (Oliv.) Diels, Paeonia lactiflora Pall. and Chuanxiong Rhizoma	HCC Mice	10 g/kg	28 d	LTF, AMPK, LTF, Beclin1, Caspase3, Caspase8, Caspase9 and Bax↑ ALT, AST, B-cell lymphoma-2, mTOR and P62↓	Firmicutes↓Bacteroidetes, Bacteroidales_unclassified, Duncaniella, Odoribacter, Parabacteroides Rikenellaceae_RC9_gut_group, Bacteroidales_unclassified, Enterococcus, Acinetobacter, Alloprevotella and Morganella↑	Regulating GM	[197]
Qijia Rougan decoction	Astragalus membranaceus (Fisch.) Bunge, Angelicae Sinensis Radix, Carapax trionycis, Ground beetle, Salvia miltiorrhiza Bunge, Carthamus tinctorius L., Semen persicae, Rhizoma sparganii, Curcumae Rhizoma and Glycyrrhiza uralensis Fisch	HF rat	28 g/kg	6 weeks	liver index, AST, ALT, ALP, IL-1β, IL-6, TNF-α, α-SMA and Collagen I↓ Claudin-1, Occludin and ZO-1↑	Bacteroidota, Prevotellaceae UCG 001, Turicibacter, Dubosiella, Faecalibaculum and Oscillibacter↓ Firmicutes, Lactobacillus, Lactobacillus johnsonii, Lactobacillus reuteri and Rikenellaceae↑	Regulate GM and maintain intestinal barrier function	[198]
Danggui Shaoyao San	Angelica sinensis (Oliv.) Diels, Poria cocos (Schw.) Wolf., Chuanxiong Rhizoma, Alisma orientale (Samuel) Juz., Atractyolodes macrocephala Koidz. and Paeonia lactiflora Pall	HF rat	2–8 g/kg/d	8 weeks	Liver index, LPS, D-lactate, TBA, CA, TCA and HDCA ↓ BSH↑	Firmicutes, Clostridium_sensu_stricto_1, Romboutsia, Monoglobus, norank_f_Coriobacteriales_Incertae_Sedis, Faecalibacterium ↑ unclassified_k_norank_d_Bacteria, Veillonella, norank_f_norank_o_Rhodospirillales, Parabacteroides and Bacteroidetes↓	Regulating GM and regulating SCFAs and BAs metabolism	[199]
Si-Ni-San	Radix glycyrrhizae Preparata, Paeonia lactiflora Pall., Aurantii Fructus Immaturus, and Radix Bupleuri	cholestatic liver injury mice	3.12–6.24 g/kg	7d	ALT, AST, TBA, TNF-α, IL-1β and IL-6↓	Bacteroidetes, Parasutterella, Eubacterium_coprostanoligenes_group, Clostridia_UCG-014, Candidatus_Arthromitus, Muribaculum and Bacteroides↑ Proteobacteria↓	Regulating GM imbalance and enhancing intestinal barrier function	[200]
Pien Tze Huang	Moschus chrysogaster, Bovis calculus, Snake bile and Notoginseng radix et rhizoma	AIH mice	117- 468 mg/kg	10d	ALT, AST, ALP, IBIL, TG, IL-17A, IL-4, IL-10↓ TP, ALB, GLB, HDL↑	Alloprevotella, Prevotellaceae-UCG-001, Dubosiella and Odoribacter↓ Lachnospiraceae-NK4A136, norank-f-norank-o-Clostridia-UCG-014 and Desulfovibrio↑	Regulating GM and memory regulatory T cells	[201]
JiGuCao capsule	Abrus melanospermus subsp. Melanospermus, Artemisia capillaris Thunb., Panax notoginseng (Burkill) F. H. Chen, Paeonia lactiflora Pall, Origanum vulgare L., Lycium chinense Mill., Ziziphus jujuba Mill., Bovis calculus artifactus and Suis fellis pulvis	NAFLD mice	1 ml / 100 g	8 weeks	ALT, AST, TG, TC, LDL, TBA, TG, TC, TBA, SCr, IL-1β, IL-6, TNF-α, IL-11, IL-22 and LPS ↓ Occludin-1, occludin, TJP1, IL-22 and SCFAs↑	unclassified_f_Lachnospiraceae, norank_f_Oscillospiraceae, Colidextribacter and Blautia ↓ Lactobacillus and Allobaculum↑	Regulating the gut liver axis and lipid metabolism	[202]

### Proteobacteria

As a dominant phylum among Gram-negative bacteria, Proteobacteria is closely associated with liver disease, particularly within the context of the gut-liver axis. In general, Proteobacteria is often considered a " bad actor " in hepatic disorders, with its increased abundance closely linked to the occurrence, progression, and severity of various liver conditions. An abnormal rise in Proteobacteria represents a core feature of GM dysbiosis and intestinal epithelial dysfunction in patients with liver disease [203]. It is not only a consequence of liver disease progression but also a significant driver of hepatic inflammation and fibrosis. Therefore, strategies aimed at modulating the GM, such as the use of probiotics, prebiotics, or fecal microbiota transplantation, to suppress the overgrowth of Proteobacteria have emerged as a highly promising new therapeutic approach for liver disease [204].

Enterobacteriaceae is an extremely important family within the Proteobacteria phylum of the human GM. It tends to overgrow more readily during gut dysbiosis and competes with other bacteria for resources [205]. For instance, studies have shown that Enterobacteriaceae can destabilize Lactobacillus and Bifidobacterium in the gut, thereby reducing host immunity and promoting the growth of harmful bacteria [206]. Moreover, via the gut-liver axis, Enterobacteriaceae acts as a key driver in the onset and development of many liver disease. The main mechanisms include: damaging the intestinal barrier, leading to bacterial translocation; releasing LPS endotoxins, which trigger hepatic inflammation; and producing harmful metabolites that directly injure hepatocytes [207]. Consequently, monitoring and regulating the GM, especially controlling the excessive proliferation of Enterobacteriaceae, has become an emerging and important direction in the management and treatment of liver disease.

Astragalus polysaccharide (APS), extracted from the roots of Astragalus membranaceus (Fisch.) Bunge, exhibits diverse biological activities including immunomodulatory, anti-inflammatory, antioxidant, antitumor, and antiviral effects [208]. Research indicates, for instance, that its immunomodulatory function involves activation of the TLR4-mediated myeloid differentiation primary response 88 (MyD88)-dependent signaling pathway [209]. Recent investigations further revealed that APS alleviates hepatic inflammation by balancing GM disturbances, specifically by decreasing the abundance of Enterobacteriaceae and increasing that of Lactobacillus and Bifidobacterium, repairing damaged jejunal villus structure, enhancing the expression of tight-junction proteins ZO-1, Occludin, and Claudin-1, and suppressing the production of inflammatory factors TNF-α, IL-1β, IL-6, and IL-17 through inhibition of the Salmonella-induced TLR4/MyD88/NF-κB signaling pathway, while also reducing serum ALT and AST levels [210].

Escherichia-Shigella, a genus under Enterobacteriaceae, is regarded as a pro-inflammatory bacterium and one of the major pathogens responsible for intestinal infections [211]. As a common gut microbe, it is closely linked to the pathogenesis and progression of liver disease such as ALD, cholestatic liver injury, MAFLD, and AIH. On one hand, Escherichia-Shigella can impair intestinal mucosal integrity, leading to LPS translocation to the liver and activation of immune responses; it may also disturb the balance of SCFAs and BAs, contributing to steatosis and inflammation [212, 213]. On the other hand, Escherichia-Shigella also encompasses many commensal strains that are harmless or even potentially beneficial in the intestines of healthy individuals. Prunella vulgaris L. (PVL) is a perennial herb first documented in "Shennong’s Classic of Materia Medica" [214]. TCM theory states that PVL has the effects of clearing the liver and improving vision, dissipating nodules, and reducing swelling, and it is frequently employed in the clinical treatment of liver disease [215]. Recent research discovered that PVL exerts a protective effect in ALD mice by ameliorating GM dysbiosis, specifically by reducing the abundance of Proteobacteria, Enterobacteriaceae, and Escherichia-Shigella, thereby improving intestinal barrier damage under alcohol-feeding conditions, decreasing LPS translocation to the liver and systemic circulation, and inhibiting the TLR4/MyD88 signaling pathway to modulate hepatic inflammation [216].

Yinzhihuang is a traditional formula known for clearing heat, detoxifying, and protecting the liver. It is used clinically to ameliorate cholestasis-induced liver injury in both adults and children. Studies have found that Yinzhihuang ameliorates cholestatic liver injury by modulating GM, specifically through reducing the abundance of Enterobacteriales, Escherichia-Shigella_unclassified, and Serratia_unclassified while increasing that of Clostridiales_unclassified, Lachnospiraceae_NK4A136_group_unclassified, and Bifidobacterium_pseudolongum. This regulation subsequently lowers hepatic BA levels and elevates fecal BA levels, thereby promoting bile excretion and maintaining intestinal mucosal barrier integrity [152]. Alisol B, an active component of Alisma orientale (Samuel) Juz., has been shown in previous studies to alleviate hyperlipidemia and reduce hepatocyte lipid accumulation and lipotoxicity in NASH mice [217, 218]. The latest research revealed that Alisol B ameliorates hepatic steatosis in the pathogenesis of MASLD by modulating GM composition, increasing the abundance of Escherichia-Shigella, Akkermansia, and Muribaculum, while decreasing the relative abundance of Desulfovibrio, Lactobacillus, and Romboutsia, and specifically inhibiting xanthine oxidase to correct purine metabolism dysregulation [219]. Other studies have identified positive correlations between Escherichia-Shigella and Prevotellaceae_UCG-001 and liver injury markers, the percentage of Th17 cells, LPS concentration, and the expression level of P-IκB/IκB in the liver. Berberine can alleviate concanavalin A-induced AIH by reducing the abundance of Prevotellaceae_UCG-001 and Escherichia-Shigella, increasing the abundance of SCFAs-producing bacteria (A. muciniphila and Lachnospiraceae_NK4A136_group), improving the intestinal barrier, regulating Treg/Th17 balance, and suppressing the activation of the LPS/TLR4/NF-κB signaling pathway [220].

Parasutterella is an important genus within the extensive Proteobacteria family, associated with inflammatory responses in the intestinal mucosa and systemic metabolic abnormalities. Research has shown that Parasutterella is linked to the development and progression of irritable bowel syndrome, as well as to chronic enteritis in IBS patients [221]. Studies indicate that a decrease in Parasutterella is a significant feature of gut dysbiosis in ALD mice and contributes to ALD progression by affecting the gut-liver axis, particularly BAs metabolism [222]. Cistanche deserticola Y. C. Ma, known in China as "desert ginseng" has attracted attention for its main active component, Cistanche deserticola polysaccharides (CP), due to their anti-inflammatory, antioxidant, and hepatoprotective properties [223–225]. Previous studies demonstrated a marked protective effect of CP on the liver in mice with alcoholic liver injury [226]. Further investigation into its underlying mechanisms revealed that CP ameliorates ALD by modulating GM diversity, reducing the relative abundance of Proteobacteria and Parasutterella while increasing that of Lactobacillus and Akkermansia, restoring intestinal barrier function, elevating the expression levels of intestinal tight-junction proteins, reducing the leakage of harmful substances such as LPS from the gut, and modulating the Nrf2/Kelch-like ECH-associated protein 1 pathway to diminish hepatic oxidative stress damage and inflammatory responses [227]. Liuweizhiji Gegen-Sangshen beverage is a health beverage derived from TCM, composed of six medicinal and edible plants. It has shown significant clinical efficacy and minimal side effects in managing alcohol-related symptoms and ALD [228]. Studies have found that Liuweizhiji Gegen-Sangshen treatment exerts a clear protective effect against ALD in mice. This is achieved by specifically modulating the GM to increase the abundance of Muribaculaceae, Alloprevotella, and Parasutterella, which enhances SCFAs production, elevates serum GLP-1 levels, and reduces GRP43 in the ileum. These changes collectively reduce hepatic lipid deposition, improve intestinal barrier function, and ultimately confer protection[229]. Sanghuangporus possesses the effect of softening the liver and dispersing nodules, demonstrating favorable hepatoprotective activity in clinical application [230]. It is reported to enhance liver function and immunity in both liver cancer patients and healthy individuals, and its product has obtained a Chinese patent(Patent Number: CN104490942B) [231]. Research has recently demonstrated that Sanghuangporus ameliorates hepatic fibrosis by remodeling GM homeostasis and correcting metabolic imbalance, primarily by increasing the abundance of Parasutterella and Catenibacterium. This leads to elevated levels of phosphatidylcholines involved in linoleic acid metabolism and reduced fatty acyls, thereby inhibiting hepatic stellate cells activation and collagen deposition [232].

Currently, research on TCM and natural products in the treatment of liver diseases through modulation of the Proteobacteria phylum is progressing actively (See Table 3). However, existing studies still have numerous limitations, with findings largely based on exploratory 16S rDNA sequencing of feces rather than on the GM itself. Nevertheless, the composition or function of the fecal microbiome differs from that of the GM, a distinction that should be taken into consideration. Therefore, further studies should be designed to elucidate the specific targets and detailed mechanisms of bacteria within the intestine, and to provide impetus for large-scale screening and clinical research of promising TCM and natural products.

**Table 3:** TCM and natural products improves liver disease by regulating proteobacteria

Metabolite name	Source	Model	Dose	Experimental period	Results	Regulatory effects on the microbiota	Mechanism of action	References
Artemisia capillaris Thunb. polysaccharide	Artemisia capillaris Thunb	Cholestatic liver injury Mice	50–100 mg/kg	14d	ALT, AST, ALP, TBA and TBIL↓	Firmicutes and Lachnospiraceae↑ Proteobacteria and Enterobacteriaceae↓	Regulating GM and activating Nrf2	[10]
Jiawei Xiaoyao San	Angelica sinensis (Oliv.) Diels, Bupleurum chinense DC., Codonopsis pilosula (Franch.) Nannf., Paeonia lactiflora Pall., Atractylodes macrocephala Koidz., Corydalis yanhusuo, Radix glycyrrhizae Preparata, Salvia miltiorrhiza Bunge, Moutan Cortex, Poria cocos(Schw.)Wolf,Coicis Semen, Gardenia jasminoides J.Ellis, Aurantii Fructus Immaturus and Citri Reticulatae Pericarpium	Liver Cancer Rat	6 ml/kg	20 weeks	Aurochenodeoxycholic acid↑ Glycoursodeoxycholic acid, glycochenodeoxycholic acid and chenodeoxycholic acid↓	Firmicutes, Lachnospiraceae↑ Bacteroidetes, Proteobacteria, Porphyromonadaceae unclassified, Bacteroides and Oscillibacter↓	Regulating GM and plasma metabolites	[233]
Kaempferol	Camellia sinensis (L.) Kuntze	Liver fibrosis mice	25–100 mg/kg	6 weeks	ALT and AST ↓ acetic acid, propionic acid and butyric acid↑	Firmicutes, Dubosiella, Lactobacillus, Bacteroides and Allobaculum↑ Bacteroidota, Proteobacteria, Faecalibaculum and Bifidobacterium↓	Restoring SCFAs levels to reduce oxidative damage	[234]
Zhenggan Huayu decoction	Astragalus mongholicus Bunge, Carapax Trionycis, Rhizoma Sparganii, Curcuma phaeocaulis Valeton, Chuanxiong Rhizoma, Salviae Miltiorrhizae Radix et Rhizoma, Hedyotis diffusa Willd. and Scutellaria barbata D. Don	Chronic hepatitis B fibrosis human	1 potion/d	24 weeks	AST, ALT, GCT, ALP, HA↓ CHE↑	Actinomycete, Proteobacteria, Bifidobacterium, Eubacteriaceae, Lactococcus, Micrococcaceae, and Rothia↑ Firmicutes, Acidaminococcus, Fusobacteriaceae, and Erysipelotrichaceae↓	Regulating GM	[235]
Phillygenin	Forsythia suspensa (Thunb.) Vahl	Liver fibrosis mice	10–40 mg/kg	29d	ALT, AST, ALP, γ-GT, HYP, HAase, LN, IV-C, PC III and TNF-α↓	Firmicutes, Ruminococcaceae_UCG-014 and Lactobacillus↑ Proteobacteria, Bacteroides and (Eubacterium)_coprostanoligenes_group↓	Regulating inflammation and GM	[236]
Si-Wu-Tang	Radix Rehmanniae Praeparata, Paeonia lactiflora Pall., Angelica sinensis (Oliv.) Diels and Chuanxiong Rhizoma	Fibrotic liver injury mice	5.2–20.8 g/kg	4 weeks	ALT, AST, ALP, TDCA↓ UDCA↑	Bacteroides, Lachnoclostridium, Parabacteroides, Lachnospiraceae and Prevotellaceae↑ Ruminococcaceae, Streptococcus, Alistipes and Rikenellaceae↓	Regulating GM and enterohepatic BAs circulation	[237]
Obacunone	Phellodendron chinense C. K. Schneid. and Dictamnus dasycarpus B. Turcz plant	MAFLD mice	5–10 mg/kg	12 weeks	TG, AST, ALT, LPS, TNF-α and IL-6 ↓	Proteobacteria, Firmicutes, Ralstonia, Dialister and Elizabethkingia↓ Bacteroidetes, Eubacterium_ventriosum_group, Eubacterium_xylanophilum_group and Akkermansia↑	Regulating GM and PPAR γ—FABP1/CD36 axis	[238]
Zexie-Baizhu Decoction	Alismatis Rhizoma and ractylodes Macrocephala Koidz	MAFLD mice	1500 mg/kg	24 weeks	ALT, AST, TC, TG and LDL-C↓	Firmicutes↓ Bacteroidetes, Proteobacteria, Akkermansia muciniphila, Bifidobacterium animalis, Lactococcus lactis, and Lactobacillus reuteri↑	Regulate GM and restore homeostasis of gut and adipose tissue	[239]
Tangshen formula	Astragalus mongholicus Bunge, Euonymus alatus (Thunb.) Sieb, Rehmannia glutinosa (Gaertn.) Libosch. ex DC., Citrus × aurantium f. aurantium, Cornus officinalis Sieb. et Zuce, Rheum palmatum L. and Panax notoginseng (Burk.) F.H. Chen	MAFLD mice	3.6 g/kg	16 weeks	IL-1β, IL-6, MCP-1 and TNF-α↓	Proteobacteria↓ Actinobacteriota, Acidobacteriota, unclassified_Bacteria, Methylomirabilota, Chloroflexi, unclassified_Atopobiaceae, Faecalibaculum, Sporosarcina, unclassified_Lachnospiraceae, Bifidobacterium, and unclassified_Eggerthellaceae↑	Regulating GM and improving metabolism	[240]

### Actinobacteria

Actinobacteria are Gram-positive bacteria comprising three major anaerobic families (Bifidobacteria, Propionibacteria, and Corynebacteria) and one aerobic family (Streptomyces). The most representative genus within the human gut Actinobacteria is Bifidobacterium, which exerts multiple beneficial effects, including anti-infective, anti-inflammatory, anti-tumor, and lipid-reducing actions [241]. On one hand, it can adhere to the intestinal mucosa to control the permeability of other bacteria, nutrients, and cellular metabolites. On the other hand, it can produce SCFAs to modulate hepatic metabolism and generate bacteriocins and other substances that help suppress the proliferation of pathogenic bacteria [242]. For example, studies have reported that administering Bifidobacterium adolescentis to NAFLD patients increases the abundance of genera such as Lactobacillus, Faecalibaculum, and Akkermansia, while improving biochemical parameters including catalase (CAT), glutathione (GSH), SOD, and MDA, thereby ameliorating immune balance, intestinal mucosal health, and liver metabolism [243]. Furthermore, research has found that Bifidobacterium pseudolongum is the species most markedly reduced in NAFLD-HCC mice. The acetate produced by this bacterium not only enters the liver via the portal vein but also binds to the G-protein-coupled receptor 43 on hepatocytes. This mechanism inhibits the IL-6/JAK1/STAT3 signaling pathway, effectively delaying the progression of NAFLD-HCC [244]. Tangshen Formula is a traditional Chinese herbal prescription used to treat metabolic diseases. Previous studies have shown that Tangshen Formula can improve diabetes-associated hyperlipidemia and liver injury [245]. Recent studies have revealed that Tangshen Formula also exerts a protective effect on NAFLD mice by increasing the relative abundance of Bifidobacterium, Faecalibaculum, and unclassified_Lachnospiraceae. This ameliorates GM dysbiosis, thereby improving the intestinal barrier and rectifying metabolic disturbances including those involving choline, glycerophospholipid, linoleic acid, and arachidonic acid, as well as mitigating chronic inflammation [240]. Additionally, Bifidobacteriales can promote the β-oxidation of linoleic acid into acetate and other SCFAs in the body by upregulating the hepatic expression of ACSL1 and FADS2 [246]. Stevia rebaudiana root polysaccharide (SRRP) is a water-soluble inulin-type polysaccharide extracted from stevia roots. Previous studies have demonstrated that SRRP administration improves GM composition in diabetic mouse models [247]. Recent studies have found that SRRP alleviates NAFLD by improving the GM, primarily through increasing the abundance of Lactobacillales and Bifidobacteriales. This promotes the elevation of circulating BAs and phosphatidylcholine, improves liver lipid metabolites, and reduces hepatic lipid accumulation [248].

Concurrently, the level of Bifidobacterium in the GM of ALD patients is also lower than that in healthy individuals. Studies have found that administering Bifidobacterium to ALD mice reduces levels of ALT, AST, TNF‑α, IL‑1β, TBA, and MDA, indicating that Bifidobacterium can ameliorate inflammation in ALD mice [249, 250]. Liuweizhiji Gegen-Sangshen oral liquid (composed of Puerariae lobatae radix, Hoveniae semen, Imperatae rhizoma, Crataegi fructus, Mori fructus, and Canarli fructus) is widely used in clinical practice for the prevention and treatment of ALD. Recent studies have found that its mechanism of action may involve modulating the GM by increasing the relative abundance of Bifidobacterium, Lactobacillus, and Bacillus while reducing that of Escherichia-Shigella and Parasutterella. This subsequently elevates ADH and ALDH activities and alcohol metabolism, restores intestinal epithelial barrier function, and thereby alleviates liver injury, hepatic steatosis, and inflammation[251].

However, the abundance of Bifidobacterium in cirrhosis patients shows variable changes. Some studies report increased Bifidobacterium abundance in animal models of cirrhosis [119], while others indicate reduced levels of Bifidobacterium in cirrhosis patients compared with healthy controls [252]. Administration of Bifidobacterium pseudocatenulatum to induced-cirrhosis rats decreased levels of TNF-α and IL-6 cytokines and reduced inducible nitric oxide synthase, indicating comprehensive improvement in both inflammation and antioxidant status [253]. A subsequent randomized clinical trial reported similar results: after using Bifidobacterium longum as a probiotic, patients showed enhanced LPS-degrading metabolism, increased butyrate levels in peripheral blood, and improved inflammatory markers [254]. Rubus chingii Hu., according to TCM theory, has the effect of nourishing the liver and kidney. Recent pharmacological studies show that extracts of Rubus chingii Hu. possess hepatoprotective, antioxidant, anti-inflammatory, and hypoglycemic effects [255]. Furthermore, research found that Rubus chingii Hu. can reduce liver injury and delay the occurrence of liver fibrosis by increasing the relative abundance of Bifidobacterium and Parabacteroides, decreasing the relative abundance of Prevotellaceae_UCG-001 and Ruminococcus_torques_group, and modulating the TGF-β/Smads signaling pathway [256]. Kaempferol is a natural flavonoid widely present in plants, with notable anti-inflammatory, antioxidant, and hepatoprotective pharmacological activities [257]. Recent research found that Kaempferol suppresses CCl₄-induced liver fibrosis progression by restoring GM diversity, reducing the relative abundance of Faecalibaculum and Bifidobacterium, while increasing that of Lactobacillus, Bacteroides, and Allobaculum, and elevating SCFAs levels in the gut and liver, thereby activating the Nrf2 signaling pathway to reduce oxidative damage [234]. Additionally, Bifidobacterium can act as a probiotic involved in trimethylamine-trimethylamine N-oxide (TMAO) metabolism to improve lipid metabolism [258]. Bie Jia Jian pill is a Chinese patent medicine containing 23 medicinal herbs, commonly used to treat cirrhosis [259]. Recent studies found that Bie Jia Jian pill can ameliorate liver injury, inhibit hepatic stellate cell activation, and alleviate cholestatic liver fibrosis by modulating the GM and its metabolites, increasing the abundance of Bifidobacterium and Lactobacillus in BDL rats, affecting the trimethylamine-flavin-containing monooxygenase 3-TMAO process, and inhibiting the TMAO-mediated PI3K/AKT signaling pathway [260].

Research on the regulation of GM dysbiosis in liver diseases by TCM and natural products is actively underway (See Table 4), which will provide new directions for exploring promising drugs and potential therapeutic strategies targeting the GM. Despite the promising outlook, existing studies have the following limitations: research on TCM formulas has largely focused on validating their overall effects, while in-depth mechanistic explanations regarding the GM-mediated metabolic transformation process of these formulas and their central role in host physiological regulation remain insufficient. Future research should incorporate advanced analytical techniques for in-depth exploration, for example, employing isotope tracing technology to establish tracking systems with high sensitivity and specificity. By labeling representative components in formulas, their biotransformation trajectories in the intestine can be traced to clarify which compounds are metabolized and utilized by specific bacterial populations. Additionally, integrating metagenomics with metabolomics to identify key functional bacteria involved in metabolic processes and their metabolites will help elucidate the dialogue mechanism underlying "TCM components-GM-host" interactions.

**Table 4:** TCM and natural products improves liver disease by regulating actinobacteria

Metabolite name	Source	Model	Dose	Experimental period	Results	Regulatory effects on the microbiota	Mechanism of action	References
Huaganjian decoction	Citri Reticulatae Pericarpium, Paeonia lactiflora Pall., Moutan Cortex, Gardeniae Fructus, Alisma orientale (Samuel) Juz. and Fritillariae Thunbergii Bulbus	NAFLD mice	0.62-2.48 g/kg	4 weeks	TG, TC, TC, TG, LDL-C, BAs, β-MCA, CYP7A1 and HMGCR↓ HDL-C, SCFAs, ZO-1, claudin-1, occludin, FXR and SHP2 ↑	Firmicutes and Actinobacteria↓ Bacteroidetes, Proteobacteria and Tenericutes↑	Regulating GM and BAs metabolism, affecting the FXR/FGF15/SHP2/CYP7A1 pathway	[112]
Prunella vulgaris L	Prunella vulgaris L	ALD Mice	150–300 mg/kg	34d	AST, ALT, TC, TG, TNF-α, IL-6, IL-1β and MCP-1↓	Actinobacteria, Coriobacteriaceae, unclassified_Clostridiales↑ Bacteroidetes, Proteobacteria, Porphyromonadaceae, S24-7 and Enterobacteriaceae↓	Improve GM imbalance and intestinal barrier damage, inhibit TLR4/MyD88 signaling pathway	[216]
Alisol B	Alisma orientale (Samuel) Juz	MAFLD mice	50–100 mg/kg	16 weeks	ALT, AST, TG, TC and LDL-C↓ HDL-C↑	Verrucomicrobiota, Bacteroidota, Akkermansia, Escherichia-Shigella and Muribaculum↑ Firmicutes, Actinobacteriota, Desulfovibrio, Streptococcus, Ligilactobacillus, Lactobacillus, Faecalibaculum, Romboutsia and Eisenbergiella↓	Regulating GM and restoring liver metabolic homeostasis	[219]
Xuemai Tong	Euscaphis japonica (Thunb. ex Roem. & Schult.) Kanitz)and Salvia miltiorrhiza Bunge	Liver fibrosis mice	0.14–0.57 g/kg,0.44–1.75 g/kg	6 weeks	ALT, AST and TMIL↓ LysoPE and LysoPC↑	Firmicutes, Lactobacillus, Desulfovibrio, Turicibacter, Allobaculum↓ Actinobacteria, Candidatus and Tenericutes↑	Regulating GM and its metabolites	[261]
Xiaobugan decoction	*Schisandra chinensis* (Turcz.) Baill., Zingiber officinale Roscoe, Neolitsea cassia (L.) Kosterm. and Ziziphus jujuba Mill	ALImice	325–650 mg/kg/d	21d	AST, ALT, ALP, MDA, Tnf-α, IL-6, IL-1β and Mcp1↓ SOD and GSH↑	Firmicutes↓ Acidobacteria, Bacteroidetes, Proteobacteria, Epsilonbacteraeota, Methanosata, Lachnospiraceae_UCG-001 and Aeriscardovia↑	Regulating GM and liver metabolism	[262]
Huangqi decoction	Astragalus membranaceus (Fisch.) Bge. var. mongholicus (Bge.) Hsiao., Glycyrrhizauralensis Fisch.and Glycyrrhiza uralensis Fisch	Cholestatic liver injury mice	2–4 g/kg	8 weeks	ALT, AST, ALP, IL-1β and TNF-α↓	Firmicutes, Proteobacteria, Enterobacteriales, Enterobacterceaeia, Gammaproteobactria, Enterobacterceaeia and Raoultella↑ Verrucomicrobia, Actinobacteria, Alistipes, Gordonibacter,, Prevotellaceae_NK3B31_group, norank_f_Bacteroides_S24-7_group, Parabacteroides_gold steinii and Lachnoclostridium↓	Regulating GM and improving intestinal barrier dysfunction	[263]
Poria cocos polysaccharides	Poria cocos (Schw.) Wolf	NAFLD mice	150 mg/kg and 300 mg/kg	4 weeks	AST, ALT, TC, TG, LDL-C and MDA ↓ HDL-C and T-AOC↑	Faecalibaculum, Escherichia_Shigella and unclassified Oscillospirales↑ Tuzzerella, Enterococcus and Staphylococcus↓	Improve liver injury, inflammation, and oxidative stress, regulate GM and NF—κ B/CCL3/CCR1 axis	[264]
Sanye tablet	Morus alba L., Nelumbo nucifera Gaertn., Crataegus pinnatifida Bunge., Salvia miltiorrhiza Bunge. and Paeonia lactiflora Pall	NAFLD mice	0.2 g/kg and 0.4 g/kg	16 weeks	ALT, AST, TG and TC↓ Cyp7a1, TUDCA and TDCA↑	Firmicutes/Bacteroidetes, Lactobacillus, and Enterococcus, Lactococcus, Streptococcus, L. murinus, E. faecalis, S. thermophilus, L. johnsonii and L. lactis↓ Blautia and Parabacteroides↑	Regulating GM and BAs metabolism to alleviate hepatic steatosis	[265]

### Verrucomicrobia

Verrucomicrobia is a phylum of Gram-negative bacteria widely distributed in natural environments and animal intestines. In recent years, it has garnered significant attention due to its positive impacts on ecology and human health, particularly the gut commensal Akkermansia muciniphila (A. muciniphila) [8]. A. muciniphila is nearly the sole representative of Verrucomicrobia in the human gut, and many 16S rRNA gene sequence surveys reporting this phylum often identify it at the species level as A. muciniphila. A recent analysis of over 2,000 A. muciniphila genomes confirmed this and indicated that A. muciniphila is the dominant species identified thus far [266].A. muciniphila is considered a promising probiotic [267]. It is a strict anaerobe, Gram-negative bacterium, constituting approximately 1%–3% of the total gut bacteria in healthy adults [268]. For Research has found that A. muciniphila has therapeutic effects on metabolic disorders [269] and immune diseases example [270], it ameliorates host metabolic disorders through coordinated effects on metabolic and inflammatory pathways, including the reduction of lipogenesis, gluconeogenesis, metabolic endotoxemia, insulin resistance, and body weight, coupled with modulation of inflammatory immune responses [271, 272]. Furthermore, A. muciniphila possesses potential for preventing many liver disease, particularly by enhancing intestinal barrier integrity and reducing systemic inflammation. Its protective role against hepatic conditions such as liver injury, ALD, NAFLD, AIH, cirrhosis, and HCC has been well-documented [115, 273–276].

Numerous studies indicate that A. muciniphila can prevent liver injury by degrading mucins, promoting goblet cell secretion of MUC2, increasing mucus layer thickness, and reducing intestinal permeability and LPS entry into the liver [277]. The hepatoprotective effect of Lycium barbarum (wolfberry) has been documented in China for thousands of years, and Lycium barbarum polysaccharide was the first reported macromolecule shown to alleviate liver fibrosis in CCl₄-treated mice [278]. Research found that peptidoglycan isolated from the fruit of Lycium barbarum exerts hepatoprotective functions by improving intestinal barrier function through increasing the abundance of A. muciniphila, modulating TGF-β/Smad7 signaling, and attenuating the degree of CCl₄-induced liver fibrosis in mice [279]. Additionally, alcohol can induce the proliferation of harmful bacteria (Enterobacteriaceae) and reduce SCFAs-producing bacteria (especially A. muciniphila), leading to a butyrate deficiency that weakens intestinal barrier repair and anti-inflammatory effects. Exosome-like nanoparticles from wolfberry can ameliorate alcoholic liver injury by remodeling the GM through altering the relative abundance of Akkermansia, Bifidobacterium, and Lactobacillus, maintaining gut homeostasis and barrier function, and increasing the expression of tight junction proteins[280]. Lonicerae japonicae Flos is a renowned plant with medicinal and edible properties, used for preventing and treating liver disease. Studies found that Lonicerae japonicae Flos can intervene in ALD by increasing the abundance of Akkermansia to maintain the intestinal barrier in rats, reducing the release of ALD-induced inflammatory factors, and regulating gene proteins associated with the PPAR/NF-κB signaling pathway [273].

Other studies report that decreased abundance of A. muciniphila may be associated with HCC progression [193]. This is because the unique structure of A. muciniphila can interact with TLR-4 to produce an anti-inflammatory response, whereas LPS-induced TLR-4-mediated inflammatory signaling is crucial for promoting liver fibrosis and HCC development [281]. Ginsenoside Rg3, an active component of Panax ginseng C. A. Mey., not only promotes tumor cell apoptosis and inhibits tumor invasion, proliferation, and angiogenesis but also suppresses metastasis and recurrence. Studies have found that nanoparticle conjugation of ginsenoside Rg3 can ameliorate HCC by modulating the GM, primarily through increasing the abundance of Bacteroidetes and A. muciniphila while reducing that of Firmicutes. This regulation decreases levels of 3-indolepropionic acid and urea, increases free fatty acids, and thereby corrects metabolic imbalance [282].

Studies have found that A. muciniphila can protect the liver by modulating the release of SCFAs that affect Treg cell differentiation or by influencing other types of signaling pathways to maintain the intestinal barrier and suppress inflammation [283]. Yinchenhao Decoction (YCHD) is a classic traditional Chinese formula originating from the Han Dynasty text, the Treatise on Cold Damage Disorders (c. 150–215 AD). It is prepared from three herbs: Artemisia caruifolia Buch., Gardenia jasminoides Ellis, and Rheum officinale Baill. Contemporary pharmacological research indicates that YCHD exhibits both liver-protecting and bile-promoting properties [284]. Recent research found that YCHD may improve AIH by increasing the abundance of SCFAs-producing bacteria (especially A. muciniphila), thereby affecting the levels of SCFAs (including butyrate and propionate), which in turn modulates the body's immune homeostasis by reversing the Th1/Tregs ratio [115].

Although these drugs targeting GM dysbiosis hold significant potential in the prevention and treatment of liver diseases (See Table 5), several limitations and challenges remain. Current studies primarily focus on basic experimental research, preliminarily uncovering potential associations within the "TCM–GM–liver" axis. However, high-quality clinical research remains relatively scarce, resulting in insufficient clinical evidence to elucidate the therapeutic effects of TCM from the perspective of the "gut–liver" axis, thereby limiting the clinical translation and broader application of relevant findings. Given this, future research should prioritize well-designed, multi-center, large-scale prospective cohort studies or randomized controlled trials. Such studies should systematically monitor dynamic changes in the composition, function, and metabolites of the GM during intervention, and correlate these with clinical efficacy indicators. This approach will help clarify, from an evidence-based medicine perspective, the scientific basis underlying TCM-mediated liver disease treatment through remodeling gut microbiota homeostasis, and provide high-level clinical evidence for precision TCM-based liver disease therapy.

**Table 5:** TCM and natural products improves liver disease by regulating actinobacteria

Metabolite name	Source	Model	Dose	Experimental period	Results	Regulatory effects on the microbiota	Mechanism of action	References
Gegen-Qinlian decoction	Pueraria montana (Lour.) Merr., Scutellaria baicalensis Georgi, Coptis chinensis French and Glycyrrhiza uralensis Fisch	NAFLD mice	3–12 g/kg/d	4 weeks	ALT, AST, ALP, TG, TC and LPS↓ ZO-1 and Occludin↑	Verrucomicrobia, Staphylococcus, Aggregatibacter, and Akkermansia↑ Adlercreutzia, Streptococcus, Desulfovibrio, Helicobacter and Anaerotruncus↓	Regulating GM-BAs axis	[274]
Galla chinensis polysaccharide	Galla chinensis	liver fibrosismice	100 mg/kg	28d	IL-1β, IL-6, TNF-α, ALT, AST and MDA ↓ IL-10, SOD, GSH-Px, T-AOC, Nrf2, HO-1 and NQO1↑	Verrucomicrobota, Akkermansia muciniphila, Dubosiella and UBA_3263↑	Activate the Nrf2 signaling pathway related to antioxidant activity and regulate GM	[276]
phenylethanol glycosides from Cistanche tubulosa	Cistanche tubulosa (Schenk) Wight	ALD mice	175–700 mg/kg	6 weeks	ALT, AST, TG, T-Bil, LPS, LBP, DAO, D-LA, TNF-α, IL-1β and IL-4↓ IL-10, IL-22 and SCFAs↑	Akkermansia ↑ Allobaculum↓	Reduce LPS levels and serum inflammatory markers, regulate GM	[285]
Huaier polysaccharides	Trametes robiniophila Murr	HCC mice	4–8 g/kg	25d	TNF-α and IL-1β↑	Firmicutes, Proteobacteria, Desulfobacterota, Dubosiella↓ Bacteroides, Verrucomicrobia, Allobaculum and Akkermansia↑	Affects GM and metabolites to alter tumor immune infiltration	[286]
Fructus Akebiae	Mucuna pruriens (L.) DC	HF mice	0.125-1.25 g/mL	4 weeks	AST, ALT, TG, T-Bil, α-SMA, collagen 1, NF-κ B, TLR4, D-turanose, Maltotetraose, Stachyose, JUN, PTGS2 and HSP90AA ↓ CASP3↑	Firmicutes, Bacteroidetes, Cyanobacteria and Proteobacteria↓ Verrucomicrobiota, Akkermansia, Colidextribacter and Lactobacillus↑	Regulating GM	[287]
Qinggan Yipi capsule	Misgurnus anguillicaudatus (Cantor), Indigo Naturalis and Bombyx Batryticatus	HF rat	0.18- 1.8 g/kg	4 weeks	ALT, AST, HA, LN, PC III, IV-C, TGF-β1, Smad2, p-Smad2, p-Smad3 and α-SMA↓	Verrucomicrobiota, norank_f__norank__o___Clostridia_UCG-014↑ Proteobacteria and Romboutsia↓	Inhibition of TGF—β 1/Smad2/3 signaling pathway and regulation of GM	[288]
probiotic-fermented Pueraria lobata	Pueraria lobata (Willd.) Ohwi	liver injury rat	5-10 ml/kg	7d	AST, ALT, ALP, TNF-α, IL-6 and IL-1β↓ CAT, SOD, GSH-Px, Nrf2, HO-1 and CAT↑	Firmicutes and Lactobacillus↑ Bacteroidota, Akkermansia, Turicibacter, Lactobacillus and Lachnospiraceae_NK4A136_group↓	Activate Nrf2 mediated signaling pathway to reduce oxidative stress and regulate GM dysbiosis	[289]
Rhein	Rheum palmatum L	ALImice	100 mg/kg	7d	ALT and AST ↓	Verrucomicrobiota, Proteobacteria, Akkermansia_muciniphila and Bacteroidaceae ↑ Lachnoclostridium, norank_o:Clostridia_UCG-014 and Roseburia↓	Improve GM disorder and regulate metabolic abnormalities and gene expression	[290]
Oridonin	Rabdosia rubescens( Hemsl.)Hara	liver injury mice	20 mg / kg	1d	ALT and AST ↓GSH and SOD↑	Akkermansiaceae and Bacteroidaceae, B. vulgatus↑	Regulating B. Vulgatus urea cycle Nrf2 axis	[291]
Si Miao Formula	Phellodendron chinense C. K. Schneid., Atractylodes Lancea (Thunb.) DC., Coix lacryma-jobi L.var.mayuen (Roman) Stapf and Achyranthes bidentata Blume	NAFLD mice	10–20 g/kg	16 weeks	ALT, AST, TC, TG, LDL-c and FBG↓	Verrucomicrobia, Proteobacteria, Akkermansia, Faecalibaculum and Caproiciproducens↑Firmicutes↓	Regulating liver lipid metabolism and GM	[292]

## Summary and prospect

The systematically compiled evidence in this article indicates that TCM and natural products exhibit beneficial ameliorative effects on various liver diseases. A commonality among these therapeutic effects is their close association with alterations in the composition and function of the GM. The GM is crucial for maintaining host metabolism and health, while dysbiosis plays a key role in the onset and progression of multiple diseases, including various liver disorders [293]. Consequently, modulating the GM to maintain its relative homeostasis, including microbial diversity, distribution, and metabolic stability, holds significant potential and clinical research value for treating a wide range of liver diseases. Traditional Chinese medicine and natural products provide a rich resource for novel drug discovery targeting the pathogenesis of various liver conditions. More importantly, a growing body of research reveals that these hepatoprotective effects do not stem from direct liver-targeted actions of TCM components but are achieved through an indirect pharmacological model: TCM acts as a 'modulator' to reshape the GM structure, which subsequently alters the profile and levels of microbiota-derived intermediary substances (e.g., metabolites such as SCFAs, BAs, TMAO, LPS, etc.). These intermediate substances serve as key effector molecules that, via gut-liver axis signaling, indirectly regulate hepatic physiological and pathological processes, exerting anti-inflammatory, anti-fibrotic, and anti-cancer effects. This indirect regulatory approach avoids the limitations of single-target drugs by modulating an entire ecosystem (the microbiota) and multiple metabolites, simultaneously influencing various signaling networks, making it more suitable for treating chronic liver diseases with multifactorial etiology. The holistic perspective and treatment-based-on-syndrome-differentiation principles of TCM align well with indirect pharmacology. The multi-component, multi-target, and multi-pathway characteristics of TCM formulas often operate through indirect mechanisms. Furthermore, since the initial site of action is in the gut, this approach may reduce off-target effects and toxicity associated with direct liver targeting. Therefore, this review not only explores the interactions between the complex GM and the pathogenesis of ALD, NAFLD, cirrhosis, ALI, AIH, and HCC, but also elaborates in detail on how TCM and natural product interventions ameliorate various liver diseases by modulating the five major bacterial phyla and their representative genera. For instance, we note that certain specific bacterial genera demonstrate favorable therapeutic effects against particular liver diseases, thereby laying a theoretical foundation for future scientific research targeting specific GM taxa for the treatment of specific liver conditions.

However, many current studies rely solely on 16S rRNA sequencing, lacking integration of metagenomics and metabolomics data, and TCM research still faces several limitations. For example, studies on TCM ameliorating liver diseases via GM modulation remain largely confined to the preclinical stage. Most mechanistic explorations reported in the literature are based on in vitro cell experiments and animal models, hindering the development of standardized protocols. Additionally, limited sample sizes and insufficient long-term follow-up in clinical trials impede comprehensive safety and efficacy assessments. Hence, future research should focus on the following aspects: 1. Conducting holistic analyses spanning from "microbiota structure" to "functional genes" and further to "metabolites." 2. Recognizing that diet, genetic background, and disease stage significantly influence microbial and metabolic responses to TCM, necessitating more refined stratification and personalized analysis. 3. Fully integrating technologies such as artificial intelligence, multi-omics, and high-throughput screening to establish and expand fingerprinting and databases for TCM and natural products, providing resources for further drug development. 4. Developing diversified therapeutic approaches to meet the demand for TCM and natural products in modulating the GM for disease treatment. 5. Advancing separation technologies for TCM and natural products, as well as improving formulation techniques and preparation procedures, to provide a foundation for targeted GM modulation rather than broad-spectrum disease treatment. Simultaneously, current research primarily focuses on correlations between gut dysbiosis and disease, and between disease outcomes and GM changes, which is insufficient. Future studies should delve deeper into the detailed mechanisms by which specific bacteria directly contribute to disease pathogenesis and explore how precise modulation of the GM can facilitate the development of precision medicine for targeted disease treatment.

In summary, TCM and natural products possesses distinct advantages and application potential in treating liver disease through GM modulation. Investigating the potential roles and regulatory mechanisms of TCM and natural products targeting the GM provides new insights and important references for developing therapeutic strategies against liver disease. Simultaneously, these explorations will enhance the understanding of how TCM and natural products modulate the GM during disease treatment, thereby promoting their clinical research and application.

## Data Availability

No datasets were generated or analysed during the current study.

## Abbreviations

AIH: Autoimmune hepatitis
ALD: Alcoholic liver disease
ALI: Acute liver injury
*A. **Muciniphila*: *Akkermansia muciniphila*
ALT: Alanine aminotransferase
AMPK: AMP-activated protein kinase
APS: Astragalus polysaccharide
AST: Aspartate aminotransferase
BAs: Bile acids
CAT: Catalase
CCl4: Carbon tetrachloride
CG: Cichorium glandulosum Boiss. & Huet.
CLI: Cholestatic liver injury
CP: Cistanche deserticola polysaccharides
DCA: Deoxycholic acid
DILI: Drug-induced liver injury
FXR: Farnesoid X receptor
GM: Gut microbiota
GRR: Glycyrrhiza uralensis Fisch
GSH: Glutathione
HC: Herpetospermum caudigerum Wall
HCC: Hepatocellular carcinoma
HDL-C: High-density lipoprotein cholesterol
HO-1: Heme oxygenase-1
IL: Interleukin
LPS: Lipopolysaccharide
MDA: Malondialdehyde
MMP: Matrix metalloproteinase
MyD88: Myeloid differentiation primary response 88
NAFLD: Nonalcoholic fatty liver disease
NASH: Non-alcoholic steatohepatitis
Nrf2: Nuclear factor erythroid 2-related factor 2
PPAR: Peroxisome proliferator-activated receptor
PVL: *Prunella vulgaris* L.
SCFAs: Short-chain fatty acids
SIBO: Small intestinal bacterial overgrowth
SNP: Sini Powder
SOD: Superoxide Dismutase
SRRP: Stevia rebaudiana root polysaccharide
TBA: Total bile acid
TCM: Traditional Chinese Medicine
TGF-β: Transforming growth factor-β
TLR4: Toll-like receptor 4
TMAO: Trimethylamine N-oxide
TNF-α: Tumor necrosis factor-alpha
UDCA: Ursodeoxycholic acid
XRZYBXD: Xie Re Zhu Yu Bu Xu Decoction
XYXD: Xiayuxue decoction
YCHD: Yinchenhao Decoction
ZO-1: Zonula occludens-1
